# Leveraging Prompt Engineering in Large Language Models for Accelerating Chemical Research

**DOI:** 10.1021/acscentsci.4c01935

**Published:** 2025-04-02

**Authors:** Feifei Luo, Jinglang Zhang, Qilong Wang, Chunpeng Yang

**Affiliations:** † Tianjin Key Laboratory of Advanced Carbon and Electrochemical Energy Storage, School of Chemical Engineering and Technology, Tianjin University, Tianjin 300350, China; ‡ Tianjin Key Lab of Machine Learning, College of Intelligence and Computing, Tianjin University, Tianjin 300350, China

## Abstract

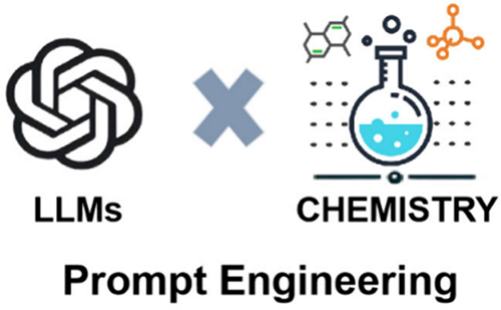

Artificial intelligence (AI) using large language models (LLMs) such as GPTs has revolutionized various fields. Recently, LLMs have also made inroads in chemical research even for users without expertise in coding. However, applying LLMs directly may lead to “hallucinations”, where the model generates unreliable or inaccurate information and is further exacerbated by limited data set and inherent complexity of chemical reports. To counteract this, researchers have suggested prompt engineering, which can convey human ideas formatively and unambiguously to LLMs and simultaneously improve LLMs’ reasoning capability. So far, prompt engineering remains underutilized in chemistry, with many chemists barely acquainted with its principle and techniques. In this Outlook, we delve into various prompt engineering techniques and illustrate relevant examples for extensive research from metal–organic frameworks and fast-charging batteries to autonomous experiments. We also elucidate the current limitations of prompt engineering with LLMs such as incomplete or biased outcomes and constraints imposed by closed-source limitations. Although LLM-assisted chemical research is still in its early stages, the application of prompt engineering will significantly enhance accuracy and reliability, thereby accelerating chemical research.

## Abstract 2

AI using large language models creates an unprecedented opportunity for chemical discovery and prompt engineering hopefully unleashes their true potential for accelerating chemical research.

## Introduction

Large Language Models (LLMs), a type of artificial intelligence (AI) designed to understand and generate human language, are trained on extensive text data sets to perform a wide range of tasks.^1−4^ They have emerged as transformative tools in various domains, including natural language processing,^5,6^ programming,^7,8^ biology^9−11^ and chemical research.^12,13^ With the ability to predict molecular property,^14^ optimize experimental designs^15−17^ and analyze vast amounts of literature,^18,19^ LLMs hold great promise for increasing the efficiency of scientific discovery in the chemistry field, especially for chemists without the expertise of coding.^20−23^ Distinct from manual approaches, LLMs can efficiently handle repetitive and time-consuming tasks, such as organizing and summarizing literature, in a more cost-effective manner. Moreover, due to their strong learning and generation capabilities, LLMs have significant potential to provide constructive scientific insights and experimental guidance, speeding up research and decision-making.^24^ In contrast to traditional models, which are typically task-specific, LLMs are highly flexible and offer higher performance in many cases. Their large-scale training data further enhances their ability to handle diverse tasks. Moreover, their user-friendly interfaces enable chemical researchers without computer science expertise to interact with them effortlessly.^25^

Recently, cutting-edge LLMs such as OpenAI-o1, Gemini 2.0 and Claude 3.5 have demonstrated significant advancements. Particularly, OpenAI-o1, which has been trained using reinforcement learning and chain-of-thought, demonstrates enhanced reasoning capabilities and leading performance across multiple benchmarks.^26^ However, directly applying them in chemical research still faces notable challenges. A key limitation is LLMs’ insufficient domain-specific expertise, which restricts their ability to provide reliable experimental guidance. Additionally, LLMs are prone to hallucination,^27^ where the model generates inaccurate or misleading information due to its reliance on broad linguistic patterns rather than domain-specific, contextually accurate reports. This challenge is further compounded by the complexity of chemical knowledge, sparse experimental data, and unstructured inputs like molecular formulas and unstructured representations, which LLMs struggle to handle accurately without specialized pretraining.

**Figure 1:**
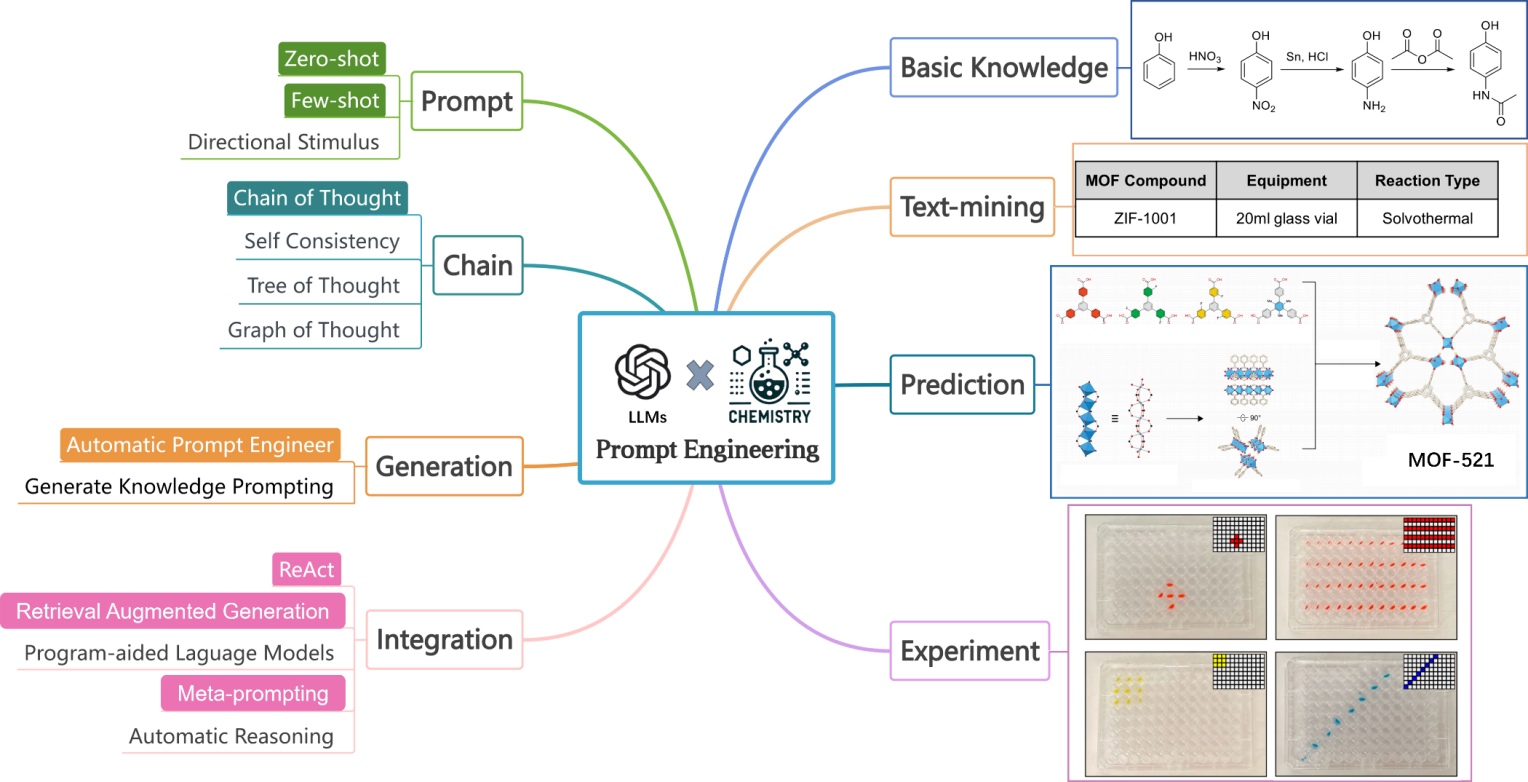
A diagram illustrating prompt engineering methods of LLMs for chemical research. Example illustrations reproduced with permission from ref (1) (Copyright 2023, Nature), ref (27) (Copyright 2023, American Chemical Society), and ref (32) (Copyright 2023, Wiley-VCH).

To counteract this, prompt engineering emerged, which enhances LLMs’ ability to better understand users’ intentions and unlock LLMs full potential to turn human aspirations into reality with remarkable effectiveness.^28^ Prompt engineering not only guides the model to correctly fulfill users’ demands but also develops a comprehensive understanding of the underlying knowledge structures essential to the specific domain. Now, prompt engineering has advanced to incorporate a variety of techniques,^29^ and we categorize them into four types: simple prompt, chain, generation and integration, which are based on the interaction between the prompt and the model (Figure 1). Currently, there are preliminary uses of prompt engineering in LLMs for chemical and materials research, such as text and image mining,^27,30^ synthesis routes prediction and optimization,^16,31−33^ aging patterns and ionic conductivity prediction^34−36^ in battery research, and automated task processing in drug discovery and materials design^1,37,38^ (Figure 1). These studies not only encompass text processing but also integrate specific chemical experiments and data analysis, providing valuable guidance and practical convenience for chemists.

Nevertheless, prompt engineering remains underutilized in the field of chemistry, with a considerable number of chemists possessing only a basic understanding of its principles and importance. Considering this, we summarize several prompt engineering technologies currently employed or potentially applicable in the chemical field. We highlight how these techniques can enhance LLMs’ chemical reasoning and accuracy through a detailed analysis of their principles, practical examples, and limitations. This Outlook offers both theoretical and practical insights, serving as a foundation for further optimization in prompt design and the broader integration of LLMs in chemistry.

## Prompt Engineering Methods

Prompt engineering, also known as in-context prompting, involves the design and optimization of input prompts to interact with LLMs for achieving the most effective outputs.^29^ The evolution of prompt engineering is closely tied to advancements in LLMs. Along increase in training data and model parameters as well as the emergence of advanced training techniques, the performance of LLM models has greatly improved.^39,40^ This approach mitigates the high costs associated with retraining and facilitates the efficient application of large-scale models. Therefore, prompt engineering is essential for facilitating interactions between LLMs and chemical research.^41^

### Zero-Shot and Few-Shot Prompting

Two common prompting strategies, zero-shot and few-shot prompting, serve different purposes in guiding the model’s performance. Zero-shot prompting presents a task without examples, depending on the model’s generalization abilities. Few-shot prompting, however, provides a few input-output examples to illustrate the task, aiding the model in understanding task specifics.^24^ For complex tasks where models may have insufficient contextual details to utilize its existing knowledge effectively, offering some representative examples enables the model to better understand the nuances of the task, ultimately enhancing its ability to generate accurate and relevant outputs for similar instances.

**Figure 2:**
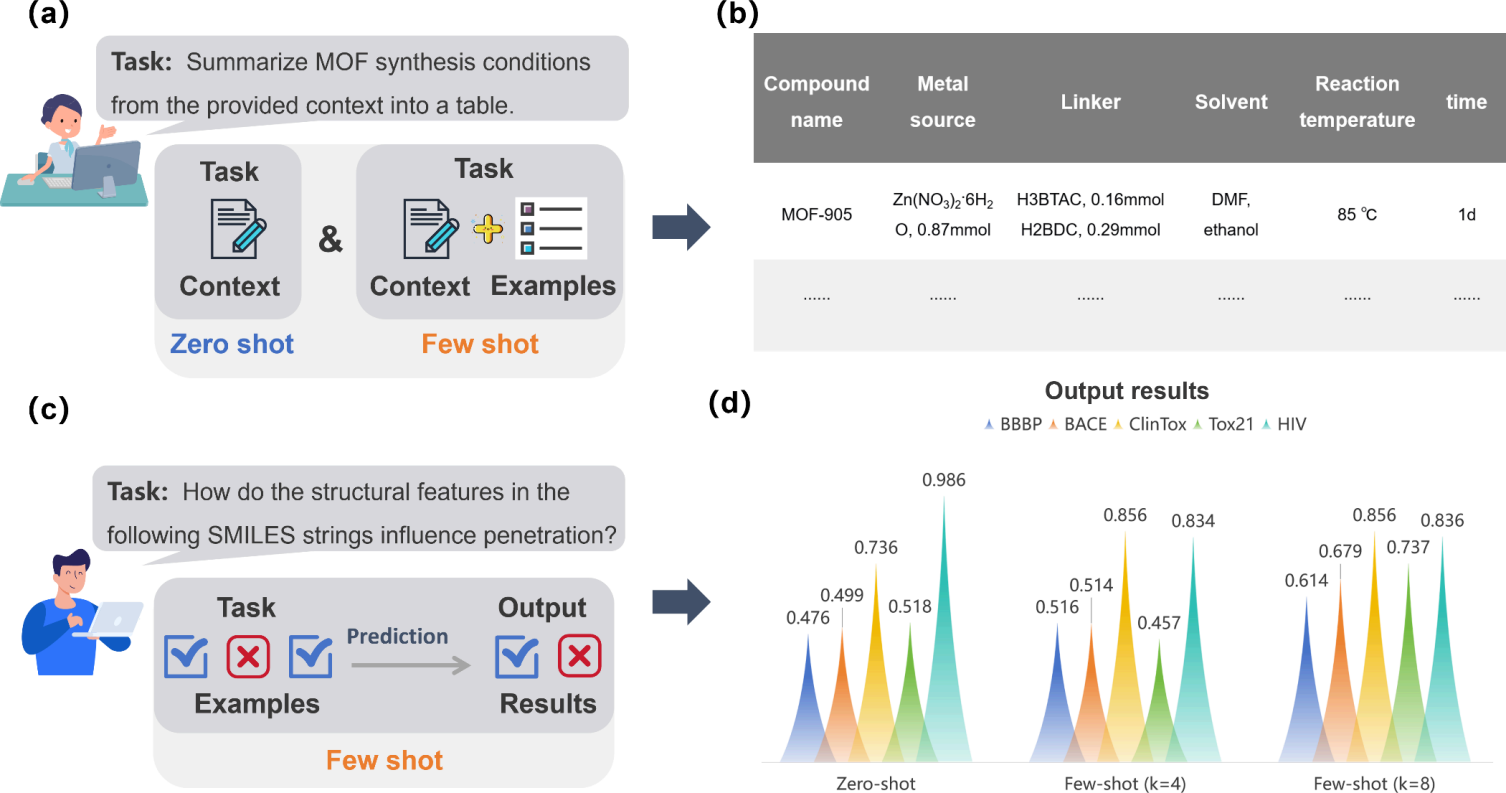
Zero-shot and few-shot prompting diagrams and testing results. (a) The simple demo of zero-shot and few-shot prompting for text mining of MOFs’ synthesis. (b) A table generated by ChatGPT which includes all the synthesis parameters of MOFs. Reproduced with permission from ref (27). Copyright 2023, American Chemical Society. (c) Demo of penetration property prediction for molecular structures represented by SMILES strings. (d) Accuracy of GPT-4 in molecular property prediction tasks, where *k* is the number of examples. BBBP, BACE, HIV Tox21, and ClinTox represent five different data sets. Data are sourced from ref (21).

Recently, Yaghi and co-workers employed the few-shot prompting and zero-shot prompting for text mining of MOF (Metal Organic Frameworks) synthesis (Figure 2a).^27^ By specifying both the input text and the desired output format, they successfully converted the given experimental sections of MOF into tables that accurately captured all the synthesis parameters, such as compound name, metal source, organic linkers, reaction temperature and duration (Figure 2b). At the same time, they found that providing four or five short examples in a few-shot prompting strategy enables ChatGPT to identify the features of synthesis paragraphs more effectively than zero-shot prompting. Guo et al. also demonstrated that few-shot prompting can significantly enhance LLMs’ performance on eight practical chemical tasks, such as name prediction, property prediction, and reaction prediction.^21^ For instance, in the task of property prediction (Figure 2c), they evaluated the accuracy of zero-shot, few-shot (*k* = 4), and few-shot (*k* = 8) approaches by using GPT-4, where *k* denotes the number of shots, across five data sets: BBBP, BACE, HIV, Tox21, and ClinTox (Figure 2d). Overall, the accuracy improves on the BBBP, and ClinTox data sets as the number of shots increases from *k* = 0, *k* = 4 to *k* = 8. However, on the HIV and Tox21 data sets, zero-shot prompting outperform those utilizing few-shot approaches. These highlight the importance of example selection, as high-quality and appropriate amounts of examples can significantly enhance the model’s reasoning abilities, while poorly chosen examples may impede its inferencing process. The effective application of few-shot prompting strategies in chemical research requires careful consideration by chemists.

### Chain-of-Thought (CoT) Prompting

**Figure 3:**
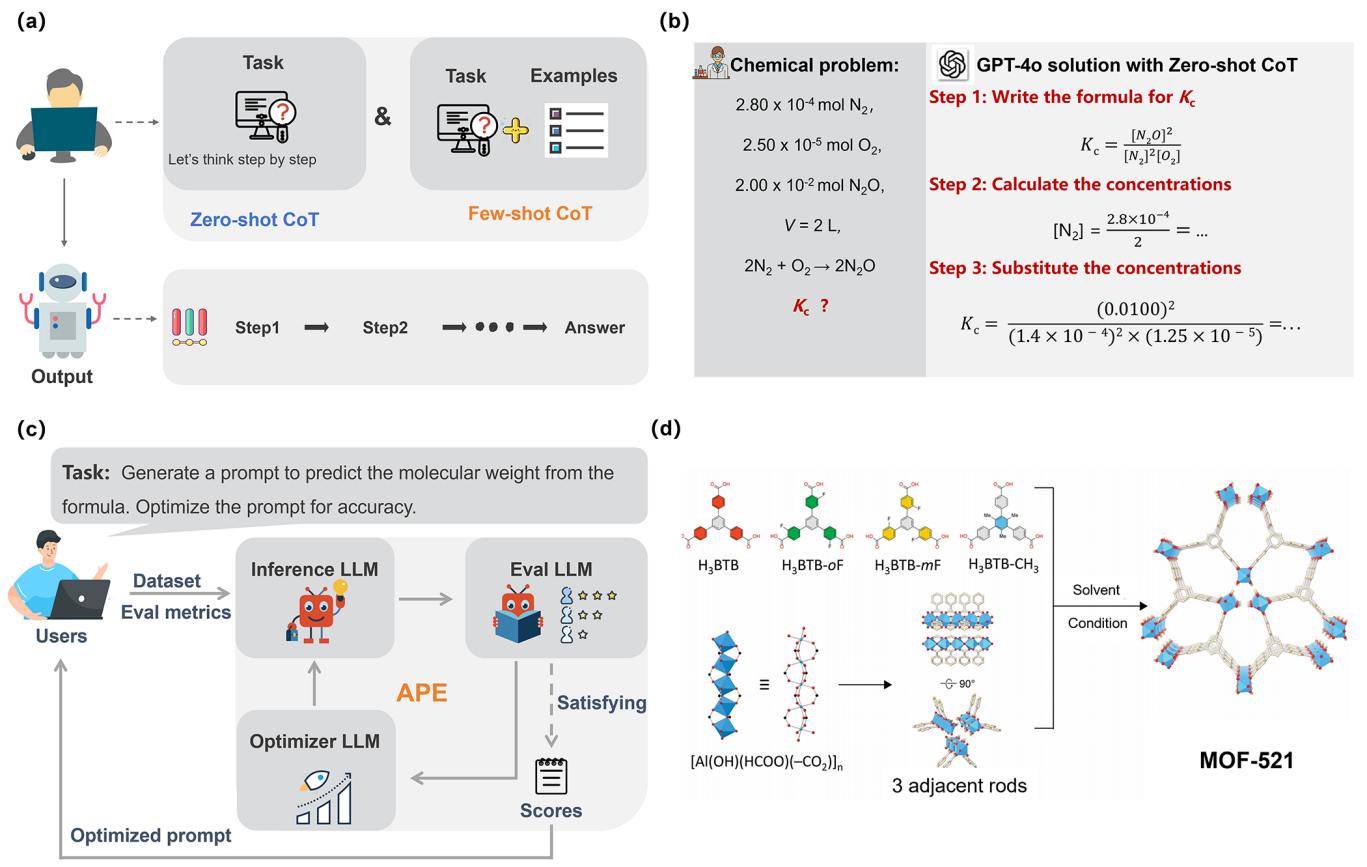
Diagrams illustrating CoT and APE. (a) The schematic diagram of CoT using zero-shot or few-shot CoT prompting. (b) The illustration of solving a complex chemistry problem (calculating *K*_c_) and the response from GPT-4o with zero-shot CoT prompting. (c) The workflowchart of APE. (d) The synthesis of MOF-521 which can be optimized by APE. Reproduced with permission from ref (32). Copyright 2023, Wiley-VCH.

While LLMs showcase considerable proficiency in basic knowledge retrieval and simple literature analysis, they face substantial limitations when tasked with complex scientific reasoning. Researchers have further found that LLMs are prone to various types of failure, often not due to an absence of domain-specific knowledge, but the lack of a robust reasoning framework to guide the processes.^42,43^ To address this challenge, Wei et al. proposed the Chain-of-Thought (CoT) prompting,^44^ which enhances traditional few-shot prompting by embedding a structured, step-by-step reasoning process in the prompts (Figure 3a). CoT prompting guides the model through a series of logical steps as demonstrated in provided examples, and encourages the model to emulate this structured reasoning, thereby producing more coherent and logically sound responses. In addition to the CoT method, Kojima et al. also introduced the zero-shot-CoT prompting.^43^ This variant utilizes a generic prompt, such as “Let’s think step by step”, to guide the model through the reasoning process without relying on specific examples. This adaptation allows models to perform reasoning tasks even when concrete examples are not available, making it a versatile tool in a broader range of contexts. So far, some advanced LLMs such as OpenAI-o1 have been trained chain-of-thought, and demonstrated strong reasoning ability, although they are not specialized logic engines. Certain complex reasoning problems, particularly those involving multistep logic or abstract thinking, may fall outside their capabilities.

The CoT prompting method has proven effective in tackling chemistry-related problems. For instance, when employing GPT-4o with zero-shot CoT prompting for equilibrium constant (*K*_c_) calculations, the model follows a three-step process (Figure 3b). First, it formulates the *K*_c_ expression based on the given chemical equation. Subsequently, it calculates the molar concentrations of the relevant species. Finally, these concentrations are substituted into the *K*_c_ expression to obtain the answer. However, the reasoning process remains imperfect. If we give GPT-4o an unbalanced chemical equation, it will encounter issues with factual hallucinations for chemical equation balancing. To solve this problem, Ouyang et al. introduced STRUCTCHEM, a CoT-based strategy that integrates a Program of Thought (PoT) and confidence verification into the CoT framework, further enhancing the reasoning accuracy of GPT-4.^45^

### Automatic Prompt Engineer (APE)

In multitask and multimodel contexts, the manual design and optimization of prompts are not only resource-intensive but also frequently constrained by rigid cognitive frameworks, ultimately hindering the models’ overall performance. To fully harness the potential of LLMs, Zhou et al. introduced the APE framework (Figure 3c), which automates the generation and optimization of prompts to enhance model performance on specific tasks.^46^ This automated approach enables LLMs efficiently to explore a diverse range of prompts, thereby maximizing the applicability and effectiveness of LLMs in various contexts. Additionally, the automated prompt optimization provided by APE can help reduce human errors by iteratively refining prompts to improve model accuracy.

So far, we have not found specific examples of APE applied in chemistry research, but some research shares similar ideas with APE in optimizing prompts. For instance, Yaghi and co-workers proposed the GPT-4 Reticular Chemist framework which demonstrates the ability to guide GPT-4 in completing MOF synthesis and optimization through prompt engineering (Figure 3d).^32^ The framework shares profound connections with APE in multiple aspects, including prompt design, iterative feedback, task decomposition, and automation goals. However, the framework relies on manually designed prompts and iterative feedback mechanisms, which limits its level of automation. In the future, by integrating APE’s automated prompt generation and optimization techniques, the efficiency and scalability of this framework could be further enhanced, promoting broader applications of LLMs in chemical discovery.

### Synergizing Reasoning + Acting (ReAct) Prompting

General prompt engineering techniques struggle with external interactions, while those designed for such tasks often lack strong reasoning. This is especially problematic in domains like chemistry, where models need both robust reasoning and external interactions. For instance, models might need to modify compound ratios based on experimental results or utilize search engines to acquire additional chemical information. ReAct prompting, proposed by Yao et al. enables both systematic reasoning and proactive engagement with external resources, overcoming the above limitations.^47^ Different from CoT prompting, ReAct examples feature not only detailed reasoning steps but also specific actions such as “searches” or “look-ups” that the model performs during the reasoning steps. By integrating external tools, ReAct prompting helps optimize chemical research workflows and support the automation of material design and complex chemical tasks. It demonstrates powerful capabilities in enhancing LLMs’ data generation, tool selection, and feedback loops, driving the intelligent advancement of chemical research.

**Figure 4:**
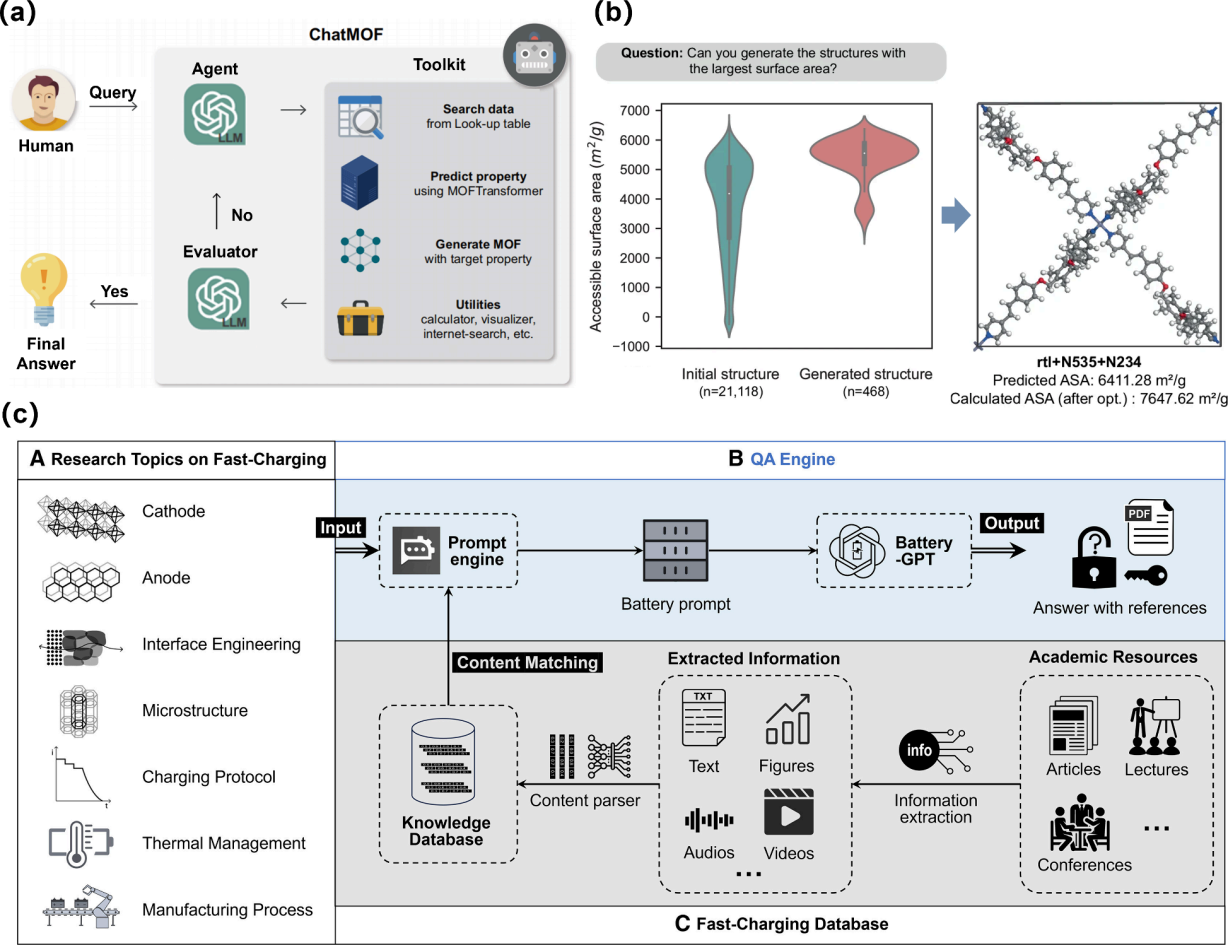
Diagram and application of ReAct and RAG. (a) The schematic image of ChatMOF, which leverages the principles of ReAct prompting. (b) The distribution of maximum surface area for initial and generated structures, and the MOF structure with the largest surface area generated by ChatMOF with optimized data. (a), (b) reproduced with permission from ref (2). Available under a CC-BY license. Copyright 2024, Springer Nature. (c) Application of LLMs with RAG for fast-charging batteries research. Reproduced with permission from ref (13). Copyright 2024, Elsevier.

Recently, Kang et al. reported the ChatMOF, which utilizes the principles of ReAct prompting to establish a highly effective framework for MOF research.^2^ Upon receiving a query about MOFs, LLMs systematically plan and select appropriate tools for data retrieval, property prediction, and MOF generation. Then, the generated MOF is evaluated. If the result is unsatisfactory, the evaluator provides feedback to the agent, prompting the process to repeat until the desired outcome is achieved (Figure 4a). For example, in response to the query “Can you generate structures with the largest surface area?″, the initial structures exhibit a wide distribution of surface areas, which gradually converge towards higher values through iterative optimization by using ChatMOF (Figure 4b). The result is the MOF structure with a predicted surface area of 6411.28 m^2^/g. After geometric optimization, the calculated surface area increases to 7647.62 m^2^/g, ranking it third highest in the CoREMOF database. This demonstrates ChatMOF’s ability to generate and refine high-performance MOFs through systematic optimization and validation.

As an advanced prompt engineering strategy, ReAct prompting excels in complex tasks but has limitations like high resource usage, reliance on external tools, and coordination challenges. Its use depends on task needs and resource constraints. For simple tasks, simpler strategies and advanced models may suffice, while for complex tasks, ReAct’s strength is significant.

### Retrieval Augmented Generation (RAG)

To handle knowledge-intensive tasks, Lewis et al. introduced a method called RAG, which can access external knowledge and help avoid hallucinations.^48^ Specifically, RAG maps the user’s input and an external knowledge database into the same vector space. By using similarity-based retrieval, it identifies the most relevant entries from the database, which are then incorporated into the prompt to augment the model’s knowledge. Different from ReAct, which relies on internal reasoning, RAG ensures reliability by grounding its responses in verified external knowledge. If ReAct resembles a detective solving a mystery through logical deduction, RAG functions as a librarian finding the right book to answer the question. For example, to explain an enzyme’s catalytic mechanism, ReAct would analyze the active site, hypothesize substrate binding, and deduce transition states. In contrast, RAG would retrieve relevant research, extract key information, and synthesize it into a clear explanation. By leveraging authoritative external knowledge, RAG minimizes conjecture and inaccuracies while facilitating the swift assimilation of new information, thereby ensuring the precision and contemporaneity of the responses.

Recently, Zhao et al. applied RAG in the BatteryGPT, a system incorporating LLMs into battery research.^13^ The core workflow of RAG consists of two key steps: knowledge retrieval and answer generation (Figure 4c). When a user submits a query, RAG first searches battery-related literature, data, or other information from a predefined knowledge base, transforming this information into vector representations for further processing. Next, RAG combines the retrieved knowledge with the user’s query to create a comprehensive prompt, which is then fed into LLMs. By leveraging the enriched contextual information, the LLMs produce a tailored response to the user’s query.

The RAG framework offers several advantages. It enables rapid retrieval and delivery of high-quality answers by drawing upon cutting-edge literature and knowledge bases, while also ensuring that the knowledge base remains dynamically updated. Additionally, it can generate hierarchical responses. For instance, when users pose a broad question related to battery anode technologies, the system provides multilayered answers ranging from general insights to more detailed technical aspects, including specific materials, methods, and references. These references are cited to ensure traceability and facilitate further exploration of the research findings. Moreover, the RAG system is more than a passive literature retrieval tool. Through integrating and analyzing information, the system can generate precise, contextually relevant answers in a short time frame, significantly enhancing research efficiency.

### Meta-prompting

**Figure 5:**
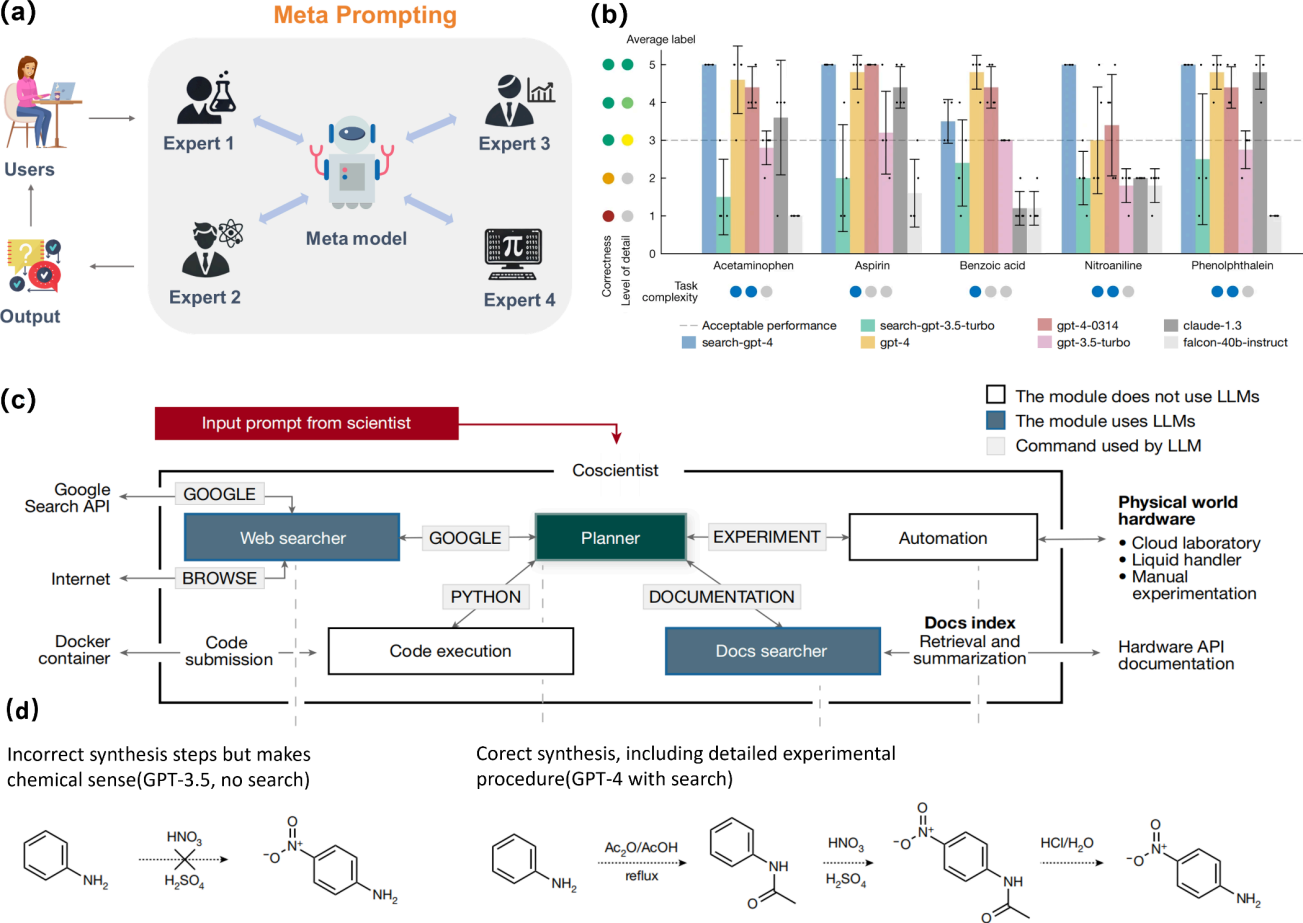
Diagram and application of meta-prompting. (a) The schematic diagram of meta-prompting; (b) Coscientist’s capabilities in chemical synthesis planning tasks. Comparison of various LLMs on compound synthesis. (c) The workflow diagram of Coscientist; (d) Two examples of generated syntheses of nitroaniline. (b)–(d) reproduced with permission from ref (1). Available under a CC-BY license. Copyright 2023, Nature.

Recently, researchers at Stanford University and OpenAI have introduced a technique called meta-prompting, which enhances the handling of complex tasks by coordinating multiple independent experts (such as LLMs or programmers).^49^ The core idea is to use a central control model to decompose a complex problem into several subtasks, assign these subtasks to specialized expert experts, and gradually interact with each expert to generate a more accurate final solution (Figure 5a). This approach leverages the expertise and diversity of multiple independent experts, thereby improving the model’s autonomy and flexibility when addressing complex challenges. Although the central model can integrate insights from multiple experts and share portions of the text with them, the experts cannot engage in direct communication. This design is intended to streamline the interaction process and ensure that the central control model remains the operational core.

Gomes and co-workers have demonstrated the development and capabilities of a multi-LLMs-based intelligent agent called Coscientist, which utilizes the principles of meta-prompting to design and conduct complex scientific experiments.^1^ With the implication of search-GPT 4 through web and documentation search, Coscientist provides detailed and accurate chemical synthesis of various substances (Figure 5b). For example, in the synthesis of nitroaniline, the planner model breaks down the task into multiple subtasks and consults an “advanced chemical expert” for assistance (Figure 5c). Followed by assigning these subtasks to other experts (such as a web searcher), Coscientist demonstrated exceptional performance in circumventing the proposal of direct nitration, which is not experimentally applicable, and at the same time, suggesting optimized reaction pathways (Figure 5d). Furthermore, in the synthesis of acetaminophen, Coscientist provided comprehensive guidance encompassing raw material selection, reaction condition optimization, and execution of experimental protocols. These instances underscore Coscientist’s efficiency and reliability in chemical synthesis planning, significantly enhancing both the success rate and overall efficiency of experimental endeavors.

Although the meta-prompting strategy has proven significantly efficient for chemical research, due to the constraints of a closed-domain system, the central model may frequently resort to apologetic language and occasionally fail to relay essential information to the experts, when handling underperforming tasks, leading to errors. Further research and optimization are needed to determine how to effectively apply the meta-prompting strategy in the chemical field.

## Summary and Outlook

In summary, prompt engineering can significantly improve the accuracy and reasoning capabilities of LLMs, thereby accelerating chemistry-related research, in various fields such as MOFs, organic synthesis, batteries, and autonomous experiments. We summarize the basic prompt engineering methods and their features and applications in Table 1. Once familiar with these, more advanced and cutting-edge approaches such as graph prompt and directional stimulus prompting can be used in more specialized and complex chemical tasks, accelerating the pace of scientific discovery.

**Table 1:** Summary of Several Prompt Engineering Methods in LLMs

Prompt Engineering	Principle	Features	Applications
Zero-shot	Directly provides task description without examples.	Simple to use, no additional data needed.	Simple classification, generation tasks (e.g., text mining of MOF synthesis^27^)
Few-shot	Provides a few examples to guide the model.	Improves model understanding of the task.	Moderately complex tasks (e.g., property prediction through SMILES^21^)
CoT	Guides the model to reason step-by-step.	Suitable for complex reasoning tasks.	Math problems, logical reasoning (e.g., calculating chemical equilibrium constants^45^)
APE	Automatically generates and optimizes prompts using the model’s own capabilities.	Reduces manual effort; may produce more effective prompts than human-designed ones.	Tasks requiring efficient prompt design.
ReAct	Solves tasks through dynamic reasoning and external actions	Suitable for multistep reasoning and external interaction tasks; improves transparency.	Complex question answering, tasks requiring external knowledge (e.g., prediction and generation of MOFs^50^)
RAG	Combines retrieval from external knowledge bases with generation to produce accurate answers.	Improves accuracy and reliability; handles tasks requiring external knowledge.	Open-domain question answering, fact-based tasks (e.g., transform words in battery research^13^)
Meta-prompting	Uses a meta-prompt to guide the model in generating specific subprompts or task decompositions.	Enhances model’s ability to understand and execute complex tasks; highly flexible.	Complex task decomposition, multistep reasoning tasks (e.g., autonomous chemical research^1^)

## Supporting Information Available

Transparent Peer Review report available (PDF)
