# Supplementary material for: Leveraging Prompt Engineering in Large Language Models for Accelerating Chemical Research
Source: ACS Cent Sci. 2025 Apr 2;11(4):511–9. doi: 10.1021/acscentsci.4c01935 (PMC12022906; doi:10.1021/acscentsci.4c01935)
Supplement: Supplementary file 1 — oc4c01935_si_001.pdf [file oc4c01935_si_001.pdf]

oc-2024-01935z.R1

Name: Peer Review Information for "Leveraging Prompt Engineering in Large Language Models for Accelerating Chemical Research"

First Round of Reviewer Comments

Reviewer: 1

Comments to the Author

The manuscript by Yang et al. presents recent progress in using intuitive language-based prompt engineering to guide large language models in assisting chemical research. This topic is very timely and addresses an emerging field. The manuscript discusses many different prompt engineering strategies, and it will read easy for the general chemistry audience, especially for experimentalist who are less familiar with AI models, to learn more about the foundations of this new field. Overall, I enjoyed reading it.

However, there are several issues that need to be addressed before publication. There are grammatical errors, a lack of proper references, and some sections are not discussed clearly, sometimes using vague terms and not giving a clear picture of the challenges being addressed. If the authors can address the following points, I believe this manuscript would make a valuable contribution to ACS Central Science as an Outlook paper.

1. For broader impact, the authors should not restrict their discussion to GPT models. Prompt engineering generally works for large language models (LLMs) such as LLaMA, Gemini, Claude, and others. I highly recommend revising the introduction to focus more on the concept of LLMs in general, rather than just GPT. Additionally, GPT is not just a single AI model; it refers to the Generative Pretrained Transformer architecture. I assume the authors are referring to the GPT series models like GPT-3.5, GPT-4, etc., which are part of the family of LLMs.

2. On page 3, line 40, the statement "It involves not only fine-tuning the model's internal mechanisms..." is conceptually incorrect. "Fine-tune" is a specific term in computer science reserved for modifying the parameters of a model, which prompt engineering does not involve. The recent arXiv article (https://arxiv.org/abs/2407.01603) discusses this distinction. Additionally, the term "internal mechanisms" is vague and may confuse readers. I suggest revising this sentence for clarity and accuracy.

3. The examples provided for LLM-guided discovery between page 3, line 57, and page 4, line 10, are too narrow in scope. Since 2023, there have been numerous works in LLM-assisted chemistry involving prompt engineering, such as:

https://doi.org/10.1038/s42256-024-00832-8

https://doi.org/10.1038/s41586-023-06792-0

https://doi.org/10.1038/s41467-024-48998-4

https://doi.org/10.1039/D3SC07012C

https://doi.org/10.1039/D3DD00239J

https://doi.org/10.1039/D4SC00924J

I strongly recommend that the authors include a more comprehensive set of references, and then clearly specifying different types of task (e.g. summarization, classification, code generation, tool-using, etc.) to capture the full scope of LLM-assisted work in chemistry. While I note that some of these works are mentioned later in the manuscript, it would be beneficial to include them earlier, perhaps in the introduction, to inform readers about what LLMs CAN do for chemists.

4. The statement "As models' capabilities have expanded, researchers have found that well-crafted prompts can effectively guide models to perform diverse tasks without the need for retraining" requires justification and literature support. What is meant by "capabilities have expanded"? Does this refer to more parameters, more training data, or higher performance on benchmark datasets? Vague terminology can leave readers unclear,

especially those unfamiliar with the field. I suggest the authors carefully revise the introduction to address the following questions:

- What are the fundamental concepts to know about LLMs?
- How do they work for chemists?
- Why use LLMs instead of traditional approaches (e.g., human-centered summarization/design) or other AI models?
- What is the current state of the art, and what needs improvement?

5. Very recently, the emergence of reasoning LLMs (e.g., O1 model) has shown even better performance without prompt engineering. The reasoning tokens allow the model to "think" before generating responses, which has proven effective in handling complex tasks. How do the authors view this development? Will this be another trend worth discussing? How will a chemist select the model to use?

6. On page 10, the manuscript discusses "ReAct Prompting." The full name should be given and explained. It appears that this approach might belong to "retrieval-augmented generation", as similar strategies sometimes have different names. If this method is indeed RAG, please clarify the differences and explain that the "searches" are conducted prior to prompting and provided to the model as input.

7. As an Outlook paper, the manuscript mainly surveys reported examples but lacks visionary, forward-looking discussion. The "Summary and Outlook" section is fairly short. It would be ideal if the authors could discuss in depth the current limitations, potential applications of LLMs that have not yet been fully exploited, and areas that could greatly benefit from the methods discussed. After reading this Outlook, readers should learn the basic prompting ideas and be inspired on how these can be employed in their own fields or to address their own questions, rather than just being told that "AI will play an increasingly important role."

8. Many of the figures are from existing literature. However, since many prompt engineering examples are text-heavy, it becomes challenging to read these extensive examples

(especially Figures 2 and 3). I suggest reorganizing or redrawing some of these figures or using Box 1, Box 2, etc., which are good for text, to present specific examples by showing the prompt, input, and output. This would give readers a better understanding of how LLMs are prompted in each scenario to achieve the desired output. Also I will suggest incoporation of Tables to make distinction on different stratergies and work reported.

Minor Comments:

1. The first sentence in the introduction is a bit confusing and lacks suitable references. It does not connect well with the rest of the introduction. Why focus on GPT (or LLM)? What makes it special? Why not other AI models?

2. Text in Figure 1 is too small and hard to read.

3. Regardless of the authors' choice of using the word "LLM" or "GPT" for this paper, the term should be plural, as more than one model/agent exists. Using the singular form may mislead readers—there are multiple AI models, and they differ significantly in performance.

4. On page 10, line 46 should be bold.

5. There have been a few comprehensive review articles on large language models for chemistry and scientific discovery. While the authors mainly focus on prompt engineering, it would be helpful to point out these existing reviews for readers who may be interested in a more detailed look.

6. While some limitations of LLMs are mentioned (e.g., hallucinations) in the introduction, I suggest adding further discussion in the manuscript on what can and cannot be done with LLMs. For example, summarization and reasoning are known strengths of LLMs, but using them to predict numerical values can be problematic (especially without fine-tuning) due to how text is tokenized and prompts are structured.

note from editor:

Please note that this paper is an Outlook article, which is a Feature Article type in the journal. Outlooks are topical, brief featured reviews that are visionary, forward-looking, accessible and of general interest to the broad readership of ACS Central Science. Please evaluate the paper in the context of this description of the article type. With this framework in mind, I hope you will agree to review this submission. I am relying on your experience, breadth and judgment to ensure that ACS Central Science continues to host only the very best content

my feelings and draft comments:

present the recent progress in using intuitive languge based prompt engineering guide AI to assist chemical research . the manuscript present nice ....

the tone it will help the audience, especially those who chemist involve less with AI model to learn more about the foundation of this new emerging field.

overall enjoy reading it. however, grammar errors, lack of propoer refence, som,

cover many examples but may not discussed in clear and convery meesgae, sometimes in vague term and not giving the pictre on what challenges were addressed. regardless, this topic is very timely

1. for boarder impact, i think the author should not resitict their discussions on GPT models - prompt engineering is generally working for large langug models like llama, gimini, claude and others. Therefore i will highly recommend revise the introduction to focus on mo the idea of "LLMs" not just GPT (also, GPT is not "an" AI model, it is a generative pretraini

transmfomer achiteture, i assume author were talking about "GPT series" like GPT-3.5, GPT-4 etc, and these should be considered as a family of LLMs)

2. page 3, line 40, conceptually, it is incorrect to say "…It involves not only fine-tuning the model's internal mechanisms"". first of all "fine-tune" is a special term in computer science that is reserved for the action involving in change the paramters, the prompt engineering does not touch any. the recent arXiv article "https://arxiv.org/abs/2407.01603" has pointed out this difference. Also the term "internal mechanisms" is a weried expression and can confuse readers.

3. the example provided for LLM-guided discovery between line 57 page 3 and line 10 page 4 is too narrow in scope, however since 2023 there have been nurousmous work in LLM-assisted chemistry with prompt engineering, such as:

https://doi.org/10.1038/s42256-024-00832-8

https://doi.org/10.1038/s41586-023-06792-0

https://doi.org/10.1038/s41467-024-48998-4

https://doi.org/10.1039/D3SC07012C

https://doi.org/10.1039/D3DD00239J

https://doi.org/10.1039/D4SC00924J

i will just name a few but strongly recommend author to make more comphrehensive reference to the scope of LLM-assited work. i do see some of the later context mention some other work, but it will be nice at intro paragph to really inform read "what LLMs can do for chemist".

4. "As models' capabilities have expanded, researchers have found that well-crafted prompts can effectively guide models to perform diverse tasks without the need for retraining." the author should have propbaly justification or literature support for this sentence. what does it mean by "capability expand", more parameters? more training data? or higher performance on benchmark Q&A dataset? in general i found more terms here can

be sometimes very vague and this sentence is just one or them, and as a reader at this point may still pocess the question of "what is a large langug model?" I think the author should carefully revise the introduction to answer the following questions:

- what are the fundamental concepts to know about LLM?

- how / in which ways do they work for chemist?

- why LLM not traidtion appoarch (e.g. human-center summary/design) / or using other AI models?

- (and maybe later in this outlook) what is the current state of the art? what possibly need to be improve

5. very recently, the emergence of reasoning LLMs (e.g. o1 model) has shown even better performance WITHOUT prompt engineering, the reasoning tokens are employed to "think" before model "say" something, and it turns out to be effective in handling complex tatsk, how will author comment on this? will this be another trend?

6. page 10 talks about "ReAct Prompting", whose full name should be given and explained. I will arugue that this likely to belong to "retrieval-augmented generation" (section 5) as people sometimes give the same strtagy (or very similar stratergy) different names. If indeed this is RAG, I would be careful here in explaining the fact that the "searches" is done prior to the prompting and given together to model for output

7. as a outlook paper, I found it mainly just surveying many examples reported but lack some visionary, forward-looking discussion. For example, the "Summary and outlook" is fairly short and it would be idealy if the author can discuss in depth what is the current limitation, and the possibilities that can be used for LLM that not yet been fully exploited, what areas can be greatly benefit from some certain methods discussed in the article. I think after reading this outlook, the reader should be able to learn the basic prompting ideas and be inspired on how this can be employed to their own field / questions, not just stating "AI will play an increasingly important role".

8. the majority of the figures are coming from exisiting literatures, however since a lot of prompt engineering examples are super text-heavy, it becomes challanges to read these overwheliming examples (especially for figure 2 and figure 3). what i will suggest is either to reorganize or redrawing some of these figures, or use "Box" to make specific examples by showing the example prompt, input and output, to give reader a better idea on each seniaor how LLM were prompted to get the desirable output.

minor:

1: some text in figure 1 is too small and hard to see

2. the first sentence in introduction is a little bit confusing and lack suitable reference. also it does not well connect with the rest of the introduction - why GPT (LLM)? what make it special? why not other AI models?

3. regardless of author's choice of using LLM or GPT, the term should be plural as in either case more than one model/agent exisits, using singular may mislead readers - there is NOT just one AI model and they each differ a lot in performance!

4. line 46 page 10 should be bold.

5. there have been a few very long and comprehensive review article in large lmauguu models for chemistry / scienctici discovery, while the author mainly focus on prompt engineering, it will be nice to point out those exisiting one to reader who may be interested in getting a more detailed look

6. some limitations of LLMs are mentioned (e.g. halluciations) in introduction, but later in the mansucirpt, i will suggest adding discussion of what can/cannot be done with LLMs. for example, summarization and reaosning is known be LLM's strnegth, but using them the predict a nuermerical number is dangerous (especially without fine-tuning) based on the way text are tokenized and prompting are given.

Reviewer: 2

Comments to the Author

This manuscript offers an overview of prompt engineering for large language models (LLMs) in the context of chemistry research. The topic is timely and important, and the starting point is appreciated. However, several areas of the manuscript could be enhanced,

particularly in terms of keeping pace with recent advancements and refining the presentation to align with the high standards of ACS Central Science. Some aspects of the discussion feel underdeveloped, and the manuscript does not fully reflect the latest developments in the field.

(1) The title, “AI-assisted,” is too general and does not adequately capture the specific focus on prompt engineering. Using the term “large language models” directly is recommended, as it provides a clearer and more precise description of the content.

(2) The manuscript primarily references models from 2023, which feels somewhat outdated for an outlook intended for publication, most likely, in 2025. Including more recent models, such as GPT-4 and OpenAI o1, and discussing their evolving capabilities would significantly enhance the manuscript’s relevance and provide a more forward-looking perspective.

(3) The example of Joblonka et al. in the few-shot learning section is described inaccurately. Joblonka et al. focus on direct fine-tuning, not few-shot learning. This distinction is crucial for maintaining technical accuracy and ensuring the integrity of the discussion.

(4) The description of GPT-4’s reasoning ability feels outdated. Models like o1, which exhibit automatic reasoning capabilities, should be highlighted to showcase the current state-of-the-art in reasoning tasks. This would provide a more up-to-date and comprehensive view of model advancements.

(5) The REACT section, while promising, lacks proper citation for the example from Kang, and the limited number of examples reduces the depth of the discussion. Expanding this section with additional examples and ensuring accurate referencing would strengthen the argument and offer a more thorough exploration of REACT’s application in chemistry research.

(6) The prompt engineering methods discussed in the manuscript are relatively basic, which makes the content accessible to chemists without extensive computer science backgrounds. While this is an advantage for a broad audience, it also limits the technical depth of the manuscript. Acknowledging this balance in the paper would be beneficial, as it can be seen as both a strength and a limitation, depending on the intended readership.

(7) There are passing comments that are not properly referenced or substantiated with adequate discussion. For example, the 3rd sentence of the first paragraph of Introduction states that “With its ability to propose novel research directions, …”. However, the reviewer

doesn’t believe that they have seen convincing examples of LLMs proposing novel directions.

Author's Response to Peer Review Comments:

Point-by-point response to the comments:

Reviewer 1

Comments: The manuscript by Yang et al. presents recent progress in using intuitive languagebased prompt engineering to guide large language models in assisting chemical research. This topic is very timely and addresses an emerging field. The manuscript discusses many different prompt engineering strategies, and it will read easy for the general chemistry audience, especially for experimentalist who are less familiar with AI models, to learn more about the foundations of this new field. Overall, I enjoyed reading it. However, there are several issues that need to be addressed before publication. There are grammatical errors, a lack of proper references, and some sections are not discussed clearly, sometimes using vague terms and not giving a clear picture of the challenges being addressed. If the authors can address the following points, I believe this manuscript would make a valuable contribution to ACS Central Science as an Outlook paper.

Author response: We thank the Reviewer for the insightful comments on our work, and we have made the following revisions according to the suggestions.

1. For broader impact, the authors should not restrict their discussion to GPT models. Prompt engineering generally works for large language models (LLMs) such as LLaMA, Gemini, Claude, and others. I highly recommend revising the introduction to focus more on the concept of LLMs in general, rather than just GPT. Additionally, GPT is not just a single AI model; it refers to the Generative Pretrained Transformer architecture. I assume

the authors are referring to the GPT series models like GPT-3.5, GPT-4, etc., which are part of the family of LLMs.

Author Response: Thank you for the valuable comments. We agree with your point that the discussion should not be restricted to GPT models but should encompass the broader concept of LLMs. To address this, we have revised the introduction to emphasize the general capabilities and applications of LLMs, not just GPT. We specifically highlight the GPT series models as examples of LLMs, and rephrased the sentence "As an AI model…".

<u>Updates in the Revised Manuscript:</u>

Line 39-41, Page 3, Revised Manuscript: Large Language Models (LLMs), a type of artificial intelligence (AI) designed to understand and generate human language, are trained on extensive text datasets to perform a wide range of tasks.

Line 55-57, Page 3, Revised Manuscript: The notable model, GPT-o1, which has been trained using reinforcement learning and chain-of-thought, demonstrates enhanced reasoning capabilities and leading performance across multiple benchmarks.

2. On page 3, line 40, the statement "It involves not only fine-tuning the model's internal mechanisms..." is conceptually incorrect. "Fine-tune" is a specific term in computer science reserved for modifying the parameters of a model, which prompt engineering does not involve.

The recent arXiv article (https://arxiv.org/abs/2407.01603) discusses this distinction. Additionally, the term "internal mechanisms" is vague and may confuse readers. I suggest revising this sentence for clarity and accuracy.

Author Response: Thank you for your insightful suggestion. It's true that prompt engineering usually does not involve "fine-tuning" which refers to model parameters. While "adjusting" might seem like a fitting replacement, we realized that the main issue lies in the overall lack of clarity in the sentence. Instead of simply substituting "fine-tuning" with "adjusting," we decided to rephrase the entire sentence for clearer expression.

Regarding the term "internal mechanisms", we accept that it lacks clarity. After further consideration, we have revised the sentence to express the idea more clearly by saying "guiding the model to correctly fulfill users' demands."

<u>Updates in the Revised Manuscript:</u>

Line 68-70, Page 4, Revised Manuscript: Prompt engineering not only guides the model to correctly fulfill users' demands, but also develops a comprehensive understanding of the underlying knowledge structures essential to the specific domain.

3. The examples provided for LLM-guided discovery between page 3, line 57, and page 4, line

10, are too narrow in scope. Since 2023, there have been numerous works in LLM-assisted chemistry involving prompt engineering, such as:

https://doi.org/10.1038/s42256-024-00832-8

https://doi.org/10.1038/s41586-023-06792-0

https://doi.org/10.1038/s41467-024-48998-4

https://doi.org/10.1039/D3SC07012C

https://doi.org/10.1039/D3DD00239J

https://doi.org/10.1039/D4SC00924J

I strongly recommend that the authors include a more comprehensive set of references, and then clearly specifying different types of task (e.g. summarization, classification, code generation, tool-using, etc.) to capture the full scope of LLM-assisted work in chemistry. While I note that some of these works are mentioned later in the manuscript, it would be beneficial to include them earlier, perhaps in the introduction, to inform readers about what LLMs CAN do for chemists.

Author Response: Thank you for the valuable comments and references. LLMs have indeed demonstrated significant potential in chemical research, such as text and image mining, property prediction and optimization of materials, and automated task processing in drug discovery and materials design, as reported in the above references. We have expanded the section to include the additional references in the Introduction that the reviewer has kindly provided, as well as other relevant studies to highlight the extensive applications of LLMs in chemistry.

Updates in the Revised Manuscript:

Line 42-45, Page 3, Revised Manuscript: With the ability to predict molecular property, optimize experimental designs and analyze vast amounts of literature, LLMs hold great promise for increasing the efficiency of scientific discovery in the chemistry field, especially for chemists without the expertise of coding.[17-20]

Line 73-79, Page 4, Revised Manuscript: Currently, there are preliminary uses of prompt engineering in LLMs for chemical and materials research, such as text and image mining,[24, 27]

synthesis routes prediction and optimization,[13, 28-30] aging patterns and ionic conductivity prediction[31-33] in battery research, and automated task processing in drug discovery and materials design[34-36](Fig. 1). These studies not only encompass text processing but also integrate specific chemical experiments and data analysis, providing valuable guidance and practical convenience for chemists.

4. The statement "As models' capabilities have expanded, researchers have found that well crafted prompts can effectively guide models to perform diverse tasks without the need for retraining" requires justification and literature support. What is meant by "capabilities have expanded"? Does this refer to more parameters, more training data, or higher performance on benchmark datasets? Vague terminology can leave readers unclear, especially those unfamiliar with the field. I suggest the authors carefully revise the introduction to address the following questions:

- What are the fundamental concepts to know about LLMs?
- How do they work for chemists?
- Why use LLMs instead of traditional approaches (e.g., human-centered summarization/design) or other AI models?
- What is the current state of the art, and what needs improvement?

Author Response: Thanks for your thoughtful and constructive feedback. For clarity, we have rephrased "As models' capabilities have expanded…" to "Along increase of training data and model parameters as well as emergence of advanced training techniques, the performance of LLM models has greatly improved." Furthermore, to provide additional clarity, model performance (in downstream tasks) improves predictably when training data

and model parameters are scaled together. Simply increasing one of these factors alone does not lead to the same degree of performance enhancement. This idea is supported by research *"Scaling Laws for Neural Language Models"* (Kaplan et al., 2020) which is called scaling law. The impact of these factors on model performance can be further understood through the scaling laws, which we also cite in the paper.

We have revised the introduction to incorporate the points you raised as follows.

(1) To address the first question regarding the fundamental concepts behind LLMs, we have expanded the Introduction to provide a clearer explanation of the key principles.

Updates in the Revised Manuscript:

Line 39-41, Page 3, Revised Manuscript: Large Language Models (LLMs), a type of artificial intelligence (AI) designed to understand and generate human language, are trained on extensive text datasets to perform a wide range of tasks.

(2) Regarding the second question on how LLMs work specifically for chemists, we have revised the Introduction to discuss how LLMs can assist in various chemical research tasks.

Updates in the Revised Manuscript:

Line 42-45, Page 3, Revised Manuscript: With the ability to predict molecular property,[11] optimize experimental designs[12-14] and analyze vast amounts of literature,[15-16] LLMs hold great promise for increasing the efficiency of scientific discovery in the chemistry field, especially for chemists without the expertise of coding.[17-20]

Line 66-68, Page 4, Revised Manuscript: To counteract this, prompt engineering emerged, which enhances LLMs' ability to better understand users' intentions and unlock LLMs full potential to turn human aspirations into reality with remarkable effectiveness.

(3) In response to the third question, we have clarified the advantages of LLMs as below.

Updates in the Revised Manuscript:

Line 45-53, Page 3, Revised Manuscript: Distinct from manual approaches, LLMs can efficiently handle repetitive and time-consuming tasks, such as organizing and summarizing literature, in a more cost-effective manner. Moreover, due to their strong learning and generation capabilities, LLMs have significant potential to provide constructive scientific insights and experimental guidance, speeding up research and

decision-making.[21] In contrast to traditional models, which are typically task-specific, LLMs are highly flexible and offer higher performance in many cases. Their large-scale training data further enhances their ability to handle diverse tasks. Moreover, their user-friendly interfaces enable chemical researchers without computer science expertise to interact with them effortlessly.[22]

(4) Finally, to address the current state of the art and areas needing improvement, we have included a discussion of the latest developments in LLMs, such as GPT-o1, Gemini 2.0, and

Claude 3.5, while also pointing out key challenges that still need to be addressed.

Updates in the Revised Manuscript:

Line 54-65, Page 3, Revised Manuscript: Recently, cutting-edge LLMs such as GPT-o1,

Gemini 2.0 and Claude 3.5 have demonstrated significant advancements. The notable model, GPT-o1, which has been trained using reinforcement learning and chain-of-thought, demonstrates enhanced reasoning capabilities and leading performance across multiple benchmarks. However, directly applying them in chemical research still faces notable challenges. A key limitation is LLMs' insufficient domain-specific expertise, which restricts their ability to provide reliable experimental guidance. Additionally, LLMs are prone to hallucination, where the model generates inaccurate or misleading information due to its reliance on broad linguistic patterns rather than domain-specific, contextually accurate reports. This challenge is further compounded by the complexity of chemical knowledge, sparse experimental data, and unstructured inputs like molecular formulas and unstructured representations, which LLMs struggle to handle accurately without specialized pre-training.

5. Very recently, the emergence of reasoning LLMs (e.g., O1 model) has shown even better performance without prompt engineering. The reasoning tokens allow the model to "think" before generating responses, which has proven effective in handling complex tasks. How do the authors view this development? Will this be another trend worth discussing? How will a chemist select the model to use?

Author Response: Thank you for your insightful questions. Indeed, the o1 model has shown significant performance improvements through reinforcement learning and chain-

of-thought reasoning, surpassing previous versions like GPT-4o. We have also mentioned this in the revised manuscript. In recent years, with the simultaneous increase in training data, model parameters, computational resources, and more sophisticated pretraining techniques, the performance of LLMs has rapidly improved. We do believe this development represents an important trend. Additionally, there are other trends, such as multimodal applications, which we discuss in the Outlook section.

Regarding model selection, while the o1 model currently offers the best performance, it comes at a high cost—its API pricing is six times that of GPT-4. In some fields, the performance gap between o1 and other models is relatively small. What's more, using prompt engineering techniques can sometimes lead to greater improvements than using newer models. Therefore, chemists should carefully consider the specific requirements of their tasks and the budget they can allocate when choosing which model to use.

Updates in the Revised Manuscript:

Line 329-346, Page 17-18, Revised Manuscript: Currently, the iteration of LLMs is progressing rapidly in computer science, creating great opportunities for AI-assisted research in chemistry. Models such as GPT-o1, Gemini 2.0, and Claude 3.5 exhibit extremely powerful performance. Nevertheless, these advanced models are often costly while their improvement over previous versions could be limited in certain fields. Despite the powerful capabilities of these models, their full potential is only unlocked when their strengths are effectively utilized. Therefore, leveraging prompt engineering can sometimes lead to much greater enhancements than simply resorting to a newer model. Thus, while staying updated on the latest LLMs, chemists are encouraged to master prompt engineering techniques with the most suitable models based on the specific needs. Looking ahead, the capabilities of LLMs are extending beyond the text-based reasoning and prediction. The integration of encoders for image, audio, and other modalities has resulted in multimodal LLMs with even greater potential. The prompt engineering techniques can also be applied to multimodal LLMs for assisting chemical studies. Considering chemical scenarios such as the diversity of molecular dynamic conformations and complexity of crystalline structures, the incorporation of prompt engineering in multimodal LLMs through interdisciplinary collaboration may become an important direction in future research. With the emergent ability of LLMs, chemists will greatly benefit from leveraging the techniques of LLMs, particularly prompt engineering, to harness LLM-based AI in driving innovation and progress in the field of chemistry.

6. On page 10, the manuscript discusses " ability Prompting." The full name should be given and explained. It appears that this approach might belong to "retrieval-augmented

generation", as similar strategies sometimes have different names. If this method is indeed RAG, please clarify the differences and explain that the "searches" are conducted prior to prompting and provided to the model as input.

Author Response: Thank you for your insightful comments. We have provided the full name of ReAct which is Synergizing Reasoning + Acting.

While ReAct and RAG share similarities, they are different methods. Specifically, ReAct enables the model to perform reasoning steps and generate actions **during its reasoning process**. In contrast, RAG involves retrieving information from an external knowledge database **before generating responses**, and it directly integrates the retrieved information into the prompt. To clarify this distinction, we have illustrated it using a simple example of an enzyme's catalytic mechanism. By comparing how ReAct and RAG would handle this example, we highlight their differences more clearly.

Updates in the Revised Manuscript:

Line 197-199, Page 10, Revised Manuscript: Different from CoT prompting, ReAct examples feature not only detailed reasoning steps but also specific actions such as "searches" or "lookups" that the model performs during the reasoning steps.

Line 223-226, Page 11, Revised Manuscript: Specifically, RAG maps the user's input and an external knowledge database into the same vector space. By using similarity-based retrieval, it identifies the most relevant entries from the database, which are then incorporated into the prompt to augment the model's knowledge.

Line 226-235, Page 11-12, Revised Manuscript: Different from ReAct, which relies on internal reasoning, RAG ensures reliability by grounding its responses in verified external knowledge. If ReAct resembles a detective solving a mystery through logical deduction, RAG functions as a librarian finding the right book to answer your question. For example, to explain an enzyme's catalytic mechanism, ReAct would analyze the active site, hypothesize substrate binding, and deduce transition states. In contrast, RAG would retrieve relevant research, extract key information, and synthesize it into a clear explanation. By leveraging authoritative external knowledge, RAG minimizes conjecture and inaccuracies while facilitating the swift assimilation of new information, thereby ensuring the precision and contemporaneity of the responses.

7. As an Outlook paper, the manuscript mainly surveys reported examples but lacks visionary, forward-looking discussion. The "Summary and Outlook" section is fairly short. It

would be ideal if the authors could discuss in depth the current limitations, potential applications of LLMs that have not yet been fully exploited, and areas that could greatly benefit from the methods discussed. After reading this Outlook, readers should learn the basic prompting ideas and be inspired on how these can be employed in their own fields or to address their own questions, rather than just being told that "AI will play an increasingly important role."

Author Response: Thank you for your insightful comments. We have made substantial revisions to enhance the outlook.

(1) As per your suggestion regarding the discussion of existing limitations, we have provided a detailed analysis of several critical challenges from both AI and chemistry perspectives. These include the issue of hallucinations, the limitations of numerical prediction, and the insufficient domain-specific knowledge in LLMs. We believe these topics are crucial for understanding the boundaries of current LLM capabilities.

(2) In terms of potential applications of LLMs that have not yet been fully exploited, we have elaborated on the emergence of multimodal LLMs and their powerful potential in advancing chemical research.

(3) We have also addressed how researchers in the chemical field can effectively employ these basic prompting ideas. Firstly, we have included a table that summarizes the basic prompt engineering methods, along with their features and applications. Secondly, by discussing the limitations of LLMs, we have demonstrated when and how different prompt engineering techniques can be effectively applied. Additionally, we have kept track of the latest developments in LLMs and guide readers in selecting the most appropriate models.

<u>Updates in the Revised Manuscript:</u>

Line 299-346, Page 15-18, Revised Manuscript: In summary, prompt engineering can significantly improve the accuracy and reasoning capabilities of LLMs, thereby accelerating chemistry-related research, in various fields such as MOFs, organic

synthesis, batteries, and autonomous experiment. We summarize the basic prompt engineering methods and their features and applications in Table 1. Once familiar with these, more advanced and cutting-edge approaches such as graph prompt and directional stimulus prompting can be used in more specialized and complex chemical tasks, accelerating the pace of scientific discovery.

Table 1. Summary of several prompt engineering methods in LLMs.

Prompt Engineering	Principle	Features	Applications
**Zero-shot**	Directly provides task description without examples.	Simple to use, no additional data needed.	Simple classification, generation tasks (e.g., text mining of MOF synthesis[24])
**Few-shot**	Provides a few examples to guide the model.	Improves model understanding of the task.	Moderately complex tasks (e.g., property prediction through SMILES[18])
**CoT**	Guides the model to reason step-by-step.	Suitable for complex reasoning tasks.	Math problems, logical reasoning (e.g., calculating chemical equilibrium constants[43])
**APE**	Automatically generates and optimizes prompts using the model's own capabilities.	Reduces manual effort; may produce more effective prompts than humandesigned ones.	Tasks requiring efficient prompt design.
**ReAct**	Solves tasks through dynamic reasoning and external actions	Suitable for multi-step reasoning and external interaction tasks; improves transparency.	Complex question answering, tasks requiring external knowledge (e.g., prediction and generation of MOFs[49])

**RAG**	Combines retrieval from external knowledge bases with generation to produce accurate answers.	Improves accuracy and reliability; handles tasks requiring external knowledge.	Open-domain question answering, fact-based tasks (e.g., transform words in battery research[10])
**Metaprompting**	Uses a meta-prompt to guide the model in generating specific sub-prompts or task decompositions.	Enhances model's ability to understand and execute complex tasks; highly flexible.	Complex task decomposition, multistep reasoning tasks (e.g., autonomous chemical research[35])

Still, the application of LLMs in assisting chemical research faces challenges from both the models and chemistry domain. A well-documented challenge of LLMs is the occurrence of hallucinations. Prompt engineering techniques can alleviate hallucinations to a certain extent. For example, few-shot prompting helps the model understand the desired interaction patterns through examples, CoT promotes deeper reasoning by leveraging the model's internal knowledge, and meta-prompting can integrate feedback from different experts to further mitigate hallucinations. Another AI-related limitation lies in numerical prediction tasks. While LLMs excel in text-based reasoning and prediction, their performance in numerical prediction tasks, particularly without fine-tuning, is often unsatisfactory. One potential way to mitigate this is by designing prompts that convert continuous numerical predictions into discrete interval predictions. This approach can reduce the complexity of continuous number generation and better align the model's output with human-level reasoning, making the task more manageable for LLMs. In addition, the complexity of chemistry further hinders the utilization of LLMs in chemical research. Most LLMs, even those with reported expertise at PhD-level like GPT-o1, primarily possess only foundational understanding of chemistry. This knowledge, though broad, does not extend deeply enough to offer effective experimental advice. As a result, LLMs may not be sufficient for complex chemical tasks. A feasible solution is fine-tuning the model with related chemical data or information, which requires not only open-source models but also significant expertise and collaboration in computer science. Another solution involves prompt engineering techniques, such as ReAct or RAG discussed earlier, which allow the model to retrieve external chemical knowledge as needed, supplement its internal knowledge, and overcome the chemistry-specific limitations.

Currently, the iteration of LLMs is progressing rapidly in computer science, creating great opportunities for AI-assisted research in chemistry. Models such as GPT-o1, Gemini 2.0, and Claude 3.5 exhibit extremely powerful performance. Nevertheless, these advanced models are often costly while their improvement over previous versions could be limited in certain fields. Despite the powerful capabilities of these models, their full potential is only unlocked when their strengths are effectively utilized. Therefore, leveraging prompt engineering can sometimes lead to much greater enhancements than simply resorting to a newer model. Thus, while staying updated on the latest LLMs, chemists are encouraged to master prompt engineering techniques with the most suitable models based on the specific needs. Looking ahead, the capabilities of LLMs are extending beyond the text-based reasoning and prediction. The integration of encoders for image, audio, and other modalities has resulted in multimodal LLMs with even greater potential. The prompt engineering techniques can also be applied to multimodal LLMs for assisting chemical studies. Considering chemical scenarios such as the diversity of molecular dynamic conformations and complexity of crystalline structures, the incorporation of prompt engineering in multimodal LLMs through interdisciplinary collaboration may become an important direction in future research. With the emergent ability of LLMs, chemists will greatly benefit from leveraging the techniques of LLMs, particularly prompt engineering, to harness LLM-based AI in driving innovation and progress in the field of chemistry.

8. Many of the figures are from existing literature. However, since many prompt engineering examples are text-heavy, it becomes challenging to read these extensive examples (especially

Figures 2 and 3). I suggest reorganizing or redrawing some of these figures or using Box 1, Box 2, etc., which are good for text, to present specific examples by showing the prompt, input, and output. This would give readers a better understanding of how LLMs are prompted in each scenario to achieve the desired output. Also I will suggest incorporation of Tables to make distinction on different strategies and work reported.

Author Response: We agree with the reviewer's comments regarding the readability challenges posed by the text-heavy examples in Figures 2 and 3, as well as other figures in the manuscript. To address this issue, we have reorganized or redraw some of these figures to improve readability and clearly show the prompt, input, and output for each scenario. However, we would like to note that since the prompt inputs are primarily text-

based, simplifying them into purely diagrammatic representations without showing the process may lead to confusion or misunderstanding among readers. Therefore, we still keep some textual content to ensure clarity. That said, we have striven to balance the amount of text with simplicity, reducing unnecessary details while maintaining the necessary information to guide readers effectively. In addition, we have also added Table 1 to summarize the core principles, advantages, and applications of seven prompt engineering techniques. This table provides a clear comparison of different strategies, enhancing the readability and understanding of the manuscript.

Minor Comments: 1. The first sentence in the introduction is a bit confusing and lacks suitable references. It does not connect well with the rest of the introduction. Why focus on GPT (or LLM)? What makes it special? Why not other AI models?

Author Response: Thank you for the valuable comments. We have added some references to the first sentence and reorganized the language to address why LLMs were chosen and what makes them special.

Updates in the Revised Manuscript:

Line 39-45, Page 3, Revised Manuscript: Large Language Models (LLMs), a type of artificial intelligence (AI) designed to understand and generate human language, are trained on extensive text datasets to perform a wide range of tasks. They have emerged as transformative tools in various domains, including natural language processing,[1-2] programming,[3-4] biology[5-7] and chemical research.[8-10] With the ability to predict molecular property,[11] optimize experimental designs[12-14] and analyze vast amounts of literature,[15-16] LLMs hold great promise for increasing the efficiency of scientific discovery in the chemistry field, especially for chemists without the expertise of coding.[17-20]

2. Text in Figure 1 is too small and hard to read.

Author Response: Thank you for the correction. We have modified Figure 1 to make the text clearer. The revised figure 1 is as follows:

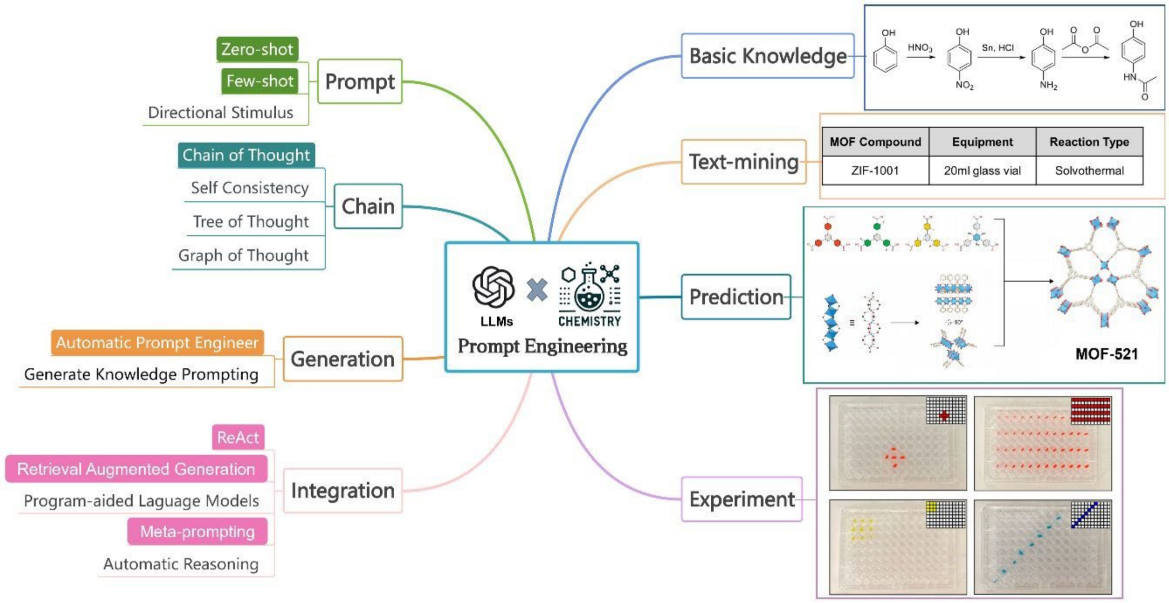

3. Regardless of the authors' choice of using the word "LLM" or "GPT" for this paper, the term should be plural, as more than one model/agent exists. Using the singular form may mislead readers—there are multiple AI models, and they differ significantly in performance.

Author Response: Thank you for the valuable comments. We fully agree with your point that "LLM" or "GPT" should be used in the plural form to accurately reflect the existence of multiple models/agents and to avoid potential misinterpretation by readers. In response to your suggestion, we have carefully reviewed the entire manuscript and revised the relevant terms to their plural forms.

4. On page 10, line 46 should be bold.

Author Response: Thank you for pointing out this issue. We have bolded this section title.

5. There have been a few comprehensive review articles on large language models for chemistry and scientific discovery. While the authors mainly focus on prompt engineering, it would be helpful to point out these existing reviews for readers who may be interested in a more detailed look.

Author Response: Thank you for the valuable suggestion. We acknowledge that there are indeed several comprehensive review articles on large language models (LLMs) in chemistry and scientific discovery. We have cited these review papers for the readers' further attention.

Updates in the Revised Manuscript:

Line 42-45, Page 3, Revised Manuscript: With the ability to predict molecular property,[11] optimize experimental designs[12-14] and analyze vast amounts of literature,[15-16] LLMs hold great promise for increasing the efficiency of scientific discovery in the chemistry field, especially for chemists without the expertise of coding.[17-20]

6. While some limitations of LLMs are mentioned (e.g., hallucinations) in the introduction, I suggest adding further discussion in the manuscript on what can and cannot be done with LLMs. For example, summarization and reasoning are known strengths of LLMs, but using them to predict numerical values can be problematic (especially without fine-tuning) due to how text is tokenized and prompts are structured.

Author Response: Thank you for the valuable suggestion. We have added the limitations of LLMs in numerical prediction tasks in the "Summary and Outlook" section.

Updates in the Revised Manuscript:

Line 313-319, Page 17, Revised Manuscript: Another AI-related limitation lies in numerical prediction tasks. While LLMs excel in text-based reasoning and prediction, their performance in numerical prediction tasks, particularly without fine-tuning, is often unsatisfactory. One potential way to mitigate this is by designing prompts that convert continuous numerical predictions into discrete interval predictions. This approach can reduce the complexity of continuous number generation and better align the model's output with human-level reasoning, making the task more manageable for LLMs.

Reviewer 2

Comments: This manuscript offers an overview of prompt engineering for large language models (LLMs) in the context of chemistry research. The topic is timely and important, and the starting point is appreciated. However, several areas of the manuscript could be enhanced, particularly in terms of keeping pace with recent advancements and refining

the presentation to align with the high standards of ACS Central Science. Some aspects of the discussion feel underdeveloped, and the manuscript does not fully reflect the latest developments in the field. **Author response:** We thank the Reviewer for the comments. This Outlook aims to offer a timely perspective, encouraging chemists to adopt and effectively utilize tools like prompt engineering to unlock the full potential of LLMs in chemistry, thereby fostering innovation and practical applications of AI in the chemical field. The development of LLMs is advancing at an unprecedented pace, and advanced models like GPT-o1 and Gemini 2.0 are not referenced in our previous manuscript. Based on our original intention and the reviewer's suggestions, we have added the required critical information and made the following revisions to the Revised Manuscript.

(1) The title, "AI-assisted," is too general and does not adequately capture the specific focus on prompt engineering. Using the term "large language models" directly is recommended, as it provides a clearer and more precise description of the content.

Author response: Thank you for your valuable suggestion on the title of our manuscript. In response to your suggestion, we have revised the title to provide a clearer and more precise description of the content by directly incorporating the term "large language models." The revised title is as follows:

"Leveraging Prompt Engineering in Large Language Models for Accelerating Chemical Research"

(2) The manuscript primarily references models from 2023, which feels somewhat outdated for an outlook intended for publication, most likely, in 2025. Including more recent models, such as GPT-4 and OpenAI o1, and discussing their evolving capabilities would significantly enhance the manuscript's relevance and provide a more forward-looking perspective.

Author Response: Thank you for your valuable feedback. We agree that including the most recent models is essential to providing a more forward-looking perspective. In our manuscript, we discussed the use of GPT-4 in Section "Chain-of-Thought Prompting" and "Metaprompting", and provided analysis of GPT-4's capabilities and its potential implications in Chemistry. To expand our discussion, we have added a substantial discussion on the current state-of-the-art LLMs, such as GPT-o1, Gemini 2.0, and Claude

3.5. Additionally, we have also briefly introduced the pretraining method and performance of GPT-o1 model.

Updates in the Revised Manuscript:

Line 54-57, Page 3, Revised Manuscript: Recently, cutting-edge LLMs such as GPT-o1,

Gemini 2.0 and Claude 3.5 have demonstrated significant advancements. The notable model, GPT-o1, which has been trained using reinforcement learning and chain-of-thought, demonstrates enhanced reasoning capabilities and leading performance across multiple benchmarks.

Line 148-152, Page 8, Revised Manuscript: So far, some advanced LLMs such as GPT-o1 have been trained chain-of-thought, and demonstrated strong reason ability, although they are not specialized logic engine. Certain complex reasoning problems, particularly those involving multi-step logic or abstract thinking, may fall outside their capabilities.

Line 320-323, Page 17, Revised Manuscript: Most LLMs, even those with reported expertise at PhD-level like GPT-o1, primarily possess only foundational understanding of chemistry. This knowledge, though broad, does not extend deeply enough to offer effective experimental advice. As a result, LLMs may not be sufficient for complex chemical tasks.

Line 329-337, Page 18, Revised Manuscript: Currently, the iteration of LLMs is progressing rapidly in computer science, creating great opportunities for AI-assisted research in chemistry. Models such as GPT-o1, Gemini 2.0, and Claude 3.5 exhibit extremely powerful performance. Nevertheless, these advanced models are often costly while their improvement over previous versions could be limited in certain fields. Despite the powerful capabilities of these models, their full potential is only unlocked when their strengths are effectively utilized. Therefore, leveraging prompt engineering can sometimes lead to much greater enhancements than simply resorting to a newer model. Thus, while staying updated on the latest LLMs, chemists are encouraged to master prompt engineering techniques with the most suitable models based on the specific needs.

(3) The example of Joblonka et al. in the few-shot learning section is described inaccurately. Joblonka et al. focus on direct fine-tuning, not few-shot learning. This distinction is crucial for maintaining technical accuracy and ensuring the integrity of the discussion.

Author Response: Thank you for pointing out the inaccuracy in our description of the work by Joblonka et al. in the few-shot learning section. In response to your feedback, we have

revised the relevant section by removing the reference to Joblonka et al. and refocusing the discussion on few-shot learning.

(4) The description of GPT-4's reasoning ability feels outdated. Models like o1, which exhibit automatic reasoning capabilities, should be highlighted to showcase the current state-of-the-art in reasoning tasks. This would provide a more up-to-date and comprehensive view of model advancements.

Author Response: Thank you for your constructive comments. We agree that highlighting the latest advancements in reasoning capabilities is important for presenting a comprehensive view of current model developments. In response, we have revised the manuscript to emphasize the reasoning abilities of GPT-o1, which, through reinforcement learning and chain-of-thought techniques, demonstrates significant advancements in various tasks. This update provides a more accurate representation of the state-of-the-art capabilities in reasoning, ensuring the manuscript remains current and forward-looking.

Updates in the Revised Manuscript:

Line 54-57, Page 3, Revised Manuscript: Recently, cutting-edge LLMs such as GPT-o1, Gemini 2.0 and Claude 3.5 have demonstrated significant advancements. The notable model, GPT-o1, which has been trained using reinforcement learning and chain-of-thought, demonstrates enhanced reasoning capabilities and leading performance across multiple benchmarks.[23]

Line 137-139, Page 7, Revised Manuscript: Researchers have further found that LLMs are prone to various types of failure, often not due to an absence of domain-specific knowledge, but the lack of a robust reasoning framework to guide its processes.

(5) The REACT section, while promising, lacks proper citation for the example from Kang, and the limited number of examples reduces the depth of the discussion. Expanding this section with additional examples and ensuring accurate referencing would strengthen the argument and offer a more thorough exploration of REACT's application in chemistry research.

Author Response: Thank you for your meticulous review and valuable feedback. Regarding the issues you raised about the ReAct section, we have made the following improvements in our subsequent revisions:

(1) Supplementing references and citations: We have carefully verified and added the relevant references for the example from Kang to ensure the accuracy and completeness of the citations. Additionally, we have organized other related literature to ensure that all cited content is properly sourced.

(2) Adding more examples: To enhance the depth of the discussion, we collect and introduce more application examples of ReAct in chemical research. These examples cover different research fields and methodologies to provide a comprehensive demonstration of ReAct's wideranging applications and potential.

Updates in the Revised Manuscript:

Line 209-216, Page 11, Revised Manuscript: For example, in response to the query "Can you generate structures with the largest surface area?", the initial structures exhibit a wide distribution of surface areas, which gradually converge towards higher values through iterative optimization by using ChatMOF (Fig. 4b). The final result is the MOF structure rtl-N535 + N234, with a predicted surface area of 6411.28 m^2/g. After geometric optimization, the calculated surface area increases to 7647.62 m^2/g, ranking it third highest in the CoREMOF database. This demonstrates ChatMOF's ability to generate and refine high-performance MOFs through systematic optimization and validation.

(6) The prompt engineering methods discussed in the manuscript are relatively basic, which makes the content accessible to chemists without extensive computer science backgrounds. While this is an advantage for a broad audience, it also limits the technical depth of the manuscript. Acknowledging this balance in the paper would be beneficial, as it can be seen as both a strength and a limitation, depending on the intended readership.

Author Response: Thank you for your insightful comment. We acknowledge that the prompt engineering methods discussed in this paper are, and have highlighted the

potential of employing more advanced approaches in the “Summary and Outlook” section.

Updates in the Revised Manuscript:

Line 302-306, Page 15, Revised Manuscript: We summarize the basic prompt engineering methods and their features and applications in Table 1. Once familiar with these, more advanced and cutting-edge approaches such as graph prompt and directional stimulus prompting can be used in more specialized and complex chemical tasks, accelerating the pace of scientific discovery.

(7) There are passing comments that are not properly referenced or substantiated with adequate discussion. For example, the 3rd sentence of the first paragraph of Introduction states that “With its ability to propose novel research directions, ...”. However, the reviewer doesn’t believe that they have seen convincing examples of LLMs proposing novel directions.

Author Response: Thank you for pointing out this issue. In light of the concern, we have revised the paragraph to remove the statement about LLMs proposing novel research directions, as this may not yet be a well-established capability. Instead, we have focused on other aspects of LLMs that are more substantiated by current research.

Updates in the Revised Manuscript:

Line 42-45, Page 3, Revised Manuscript: With the ability to predict molecular property, optimize experimental designs and analyze vast amounts of literature, LLMs hold great promise for increasing the efficiency of scientific discovery in the chemistry field, especially for chemists without the expertise of coding.

FORMATTING NEEDS

PULL QUOTE (OUTLOOKS): Please select 3 - 4 quotes from your submission that you would like highlighted in your paper. The quotes should be one sentence-long, unique to the submission and not from previously cited work. Please list your quotes at the end of the manuscript file.

Author Response: Thank you for your valuable feedback. We have selected 4 quotes from the manuscript and listed the quotes at the end of the manuscript file.

1. LLMs hold great promise for increasing the efficiency of scientific discovery in the chemistry field, especially for users without the expertise of coding.
2. Prompt engineering can significantly improve the accuracy and reasoning capabilities of LLMs, thereby accelerating chemistry-related research, in various fields such as MOFs, organic synthesis, batteries, and autonomous experiment.
3. Few-shot prompting helps the model understand the desired interaction patterns through examples, CoT promotes deeper reasoning by leveraging the model's internal knowledge, and meta-prompting can integrate feedback from different experts to further mitigate hallucinations.
4. The prompt engineering techniques can also be applied to multimodal LLMs for assisting chemical studies.

TOC GRAPHIC: The TOC graphic should be closely tied to the science in the article, so photos of people or nonscientific images such as cartoon characters, clip art and anthropomorphized molecules are not acceptable. Previously published images must also be avoided. Please see this link for the TOC guidelines:

https://pubsapp.acs.org/paragonplus/submission/toc_abstract_graphics_guidelines.pdf

Author Response: We have updated the graphic as follows:

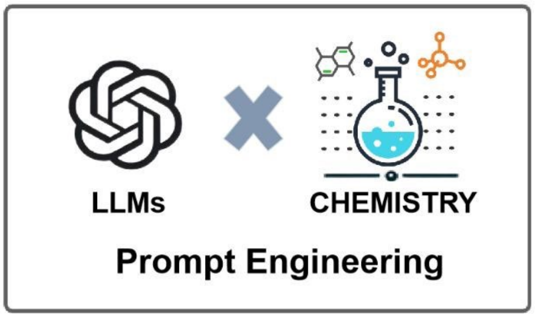

SYNOPSIS MISSING: ACS Central Science requires a brief synopsis. The synopsis should be no more than 200 characters (including spaces) and should reasonably correlate with the Table of Contents (TOC) graphic. The synopsis is intended to explain the importance of the article to a broader readership across the sciences. Please place your synopsis in the manuscript file after the TOC graphic.

Author Response: As requested, we have added a concise synopsis to the manuscript file, placed after the TOC graphic. The added synopsis reads as follows:

AI using large language models creates an unprecedented opportunity for chemical discovery and prompt engineering hopefully unleashes their true potential for accelerating chemical research.

oc-2024-01935z.R2

Name: Peer Review Information for "Leveraging Prompt Engineering in Large Language Models for Accelerating Chemical Research"

Second Round of Reviewer Comments

Reviewer: 1

Comments to the Author

After carefully reading it, I see considerable improvement in this revised manuscript on using LLMs for chemical research. The authors have done well to integrate newer research updates, clarify the role of models in chemical research, and expand the Outlook discussion to include both practical limitations and potential future directions. Below are some minor modifications that I believe will further strengthen the manuscript:

1. A minor clarification is needed: on page 3, line 55, and in other places, the manuscript refers to "GPT-o1," which is not a model name. Generally, it is referred to as "o1" or

"OpenAI-o1," and there are several variants, including "o1-mini" and "o3." The authors should refine their phrasing to avoid confusion for the readers.

2. I am uncertain whether Gemini 2.0 and Claude 3.5 explicitly use reinforcement learning and chain-of-thought reasoning for their reasoning capabilities. Unless the authors have clear technical references from these non-open-source models, I recommend avoiding definitive language about their training methodologies to prevent misleading readers. Instead, they could use terms like "may be based on" and include proper references for accuracy.

3. Another crucial aspect the authors mentioned in their response is the pricing difference between these models, which touches on a broader and even more critical issue: energy consumption during model training and API usage. I strongly encourage the authors to mention this in the Outlook section, ensuring that readers understand that there is no "free lunch" when using these models, whether open-source or proprietary. It would also be valuable to discuss potential advancements in reducing energy consumption and increasing efficiency in future LLMs.

4. Since the paper's primary focus is prompt engineering, I highly recommend adding a dedicated box or scheme to illustrate actual prompts used for all (or most) methods in Table 1. This would be particularly beneficial for chemistry researchers new to chatbots and LLMs. While Figure 2 includes examples of zero-shot and few-shot prompting, a more comprehensive and structured comparison would be helpful. A concrete example could be provided for each method, showing how a specific chemistry-related task is addressed using different prompting strategies. A useful reference for structuring such a comparison is https://doi.org/10.1038/s41578-025-00772-8, which presents prompt-based differences in a clear and instructive manner.

5. Regarding my earlier comment #6, I appreciate that the authors have addressed key limitations, particularly in discussing alternative approaches like directed prediction. If they can include examples to illustrate this, that would be even better, but the current discussion is already a strong improvement. Additionally, it is worth considering how function-calling or tool-using capabilities in LLMs can be leveraged to access external prediction tools (e.g., Python-based mathematical calculations, trained ML models, or

computational chemistry software). Instead of having LLMs directly generate predictions, they could be prompted to determine appropriate input parameters for external tools, retrieve results, and interpret them. This would be a valuable point to introduce in the discussion of prompt engineering's future applications.

Overall, I see that the manuscript has improved significantly, and I am sure addressing above additional minor points would further enhance its clarity and usefulness to the intended chemistry audience.

Reviewer: 2

Comments to the Author

The authors have addressed all my comments adequately.

Author's Response to Peer Review Comments:

Point-by-point response to the comments

Reviewer 1

Comments:

After carefully reading it, I see considerable improvement in this revised manuscript on using LLMs for chemical research. The authors have done well to integrate newer research updates, clarify the role of models in chemical research, and expand the Outlook discussion to include both practical limitations and potential future directions. Below are some minor modifications that I believe will further strengthen the manuscript.

Author response: We thank the Reviewer for the insightful comments on our work, and we have made the following revisions according to the suggestions.

1. A minor clarification is needed: on page 3, line 55, and in other places, the manuscript refers to "GPT-o1," which is not a model name. Generally, it is referred to as "o1" or "OpenAI-

o1," and there are several variants, including "o1-mini" and "o3." The authors should refine their phrasing to avoid confusion for the readers.

Author Response: Thank you for pointing this out. We realized that the term “GPT-o1” was used in error and could lead to confusion for readers. On page 3, line 55, and in all other sentences, we have replaced “GPT-o1” with the correct term “o1” or “OpenAI-o1” as appropriate.

2. I am uncertain whether Gemini 2.0 and Claude 3.5 explicitly use reinforcement learning and chain-of-thought reasoning for their reasoning capabilities. Unless the authors have clear technical references from these non-open-source models, I recommend avoiding definitive language about their training methodologies to prevent misleading readers. Instead, they could use terms like "may be based on" and include proper references for accuracy.

Author Response: Thanks for your suggestion. We understand that the training methods of Gemini 2.0 and Claude 3.5 should be described with more caution. However, in the sentences

"Recently, cutting-edge LLMs such as OpenAI-o1, Gemini 2.0, and Claude 3.5 have demonstrated significant advancements. The notable model, OpenAI-o1, which has been trained using reinforcement learning and chain-of-thought, demonstrates enhanced reasoning capabilities and leading performance across multiple benchmarks", we did not intend to state that Gemini 2.0 and Claude 3.5 use reinforcement learning and chain-of-thought methods. Instead, we aimed to highlight the significant advancements these models have made in their capabilities and performance, with a particular emphasis on OpenAI-o1, which has been trained using these techniques. To avoid ambiguity, we have modified the sentences.

Updates in the Revised Manuscript:

Line 54-57, Page 3, Revised Manuscript: "Recently, cutting-edge LLMs such as OpenAI-o1, Gemini 2.0, and Claude 3.5 have demonstrated significant advancements. Particularly, OpenAIo1, which has been trained using reinforcement learning and chain-of-thought, demonstrates enhanced reasoning capabilities and leading performance across multiple benchmarks",

3. Another crucial aspect the authors mentioned in their response is the pricing difference between these models, which touches on a broader and even more critical issue: energy consumption during model training and API usage. I strongly encourage the authors to mention this in the Outlook section, ensuring that readers understand that there is no "free lunch" when using these models, whether open-source or proprietary. It would also be valuable to discuss potential advancements in reducing energy consumption and increasing efficiency in future LLMs.

Author Response: Thank you for the valuable comments. We agree that energy consumption during model training and API usage is a crucial issue. We addressed the pricing differences between these models in the manuscript. We have now revised the text to more explicitly highlight the implications of consumption, ensuring that readers understand the trade-offs involved.

Updates in the Revised Manuscript:

Line 333-346, Page 17, Revised Manuscript: For example, while the o1 model currently offers the best performance, it comes at a steep price—its application programming interface (API) pricing is six times higher than that of GPT-4, and the energy consumption during the training process of o1 is undoubtedly much larger. In certain domains, the performance gap between o1 and other models is relatively small.

4. Since the paper's primary focus is prompt engineering, I highly recommend adding a dedicated box or scheme to illustrate actual prompts used for all (or most) methods in Table 1. This would be particularly beneficial for chemistry researchers new to chatbots and LLMs. While Figure 2 includes examples of zero-shot and few-shot prompting, a more comprehensive and structured comparison would be helpful. A concrete example could be provided for each method, showing how a specific chemistry-related task is addressed using different prompting

strategies. A useful reference for structuring such a comparison is https://doi.org/10.1038/s41578-025-00772-8, which presents prompt-based differences in a clear and instructive manner.

Author Response: Thank you for your valuable suggestion and the newest reference. We agree that a dedicated illustration of prompts would be helpful. However, we find that presenting the text-heavy prompts in a combined figure would make the text overly cumbersome and to some extent repeating with Table 1.

The prompt methods are schematically presented with examples and dispersed in the figures of each section. We have modified the schemes into unified style for clarity, which allows readers to easily compare the prompt processes across different methods. Additionally, the schemes for the prompt engineering methods can be easily combined into a single figure as shown below for comparing and understanding, together with Table 1. We hope this approach strikes a good balance between readability and conciseness, helping readers better understand the prompt methods.

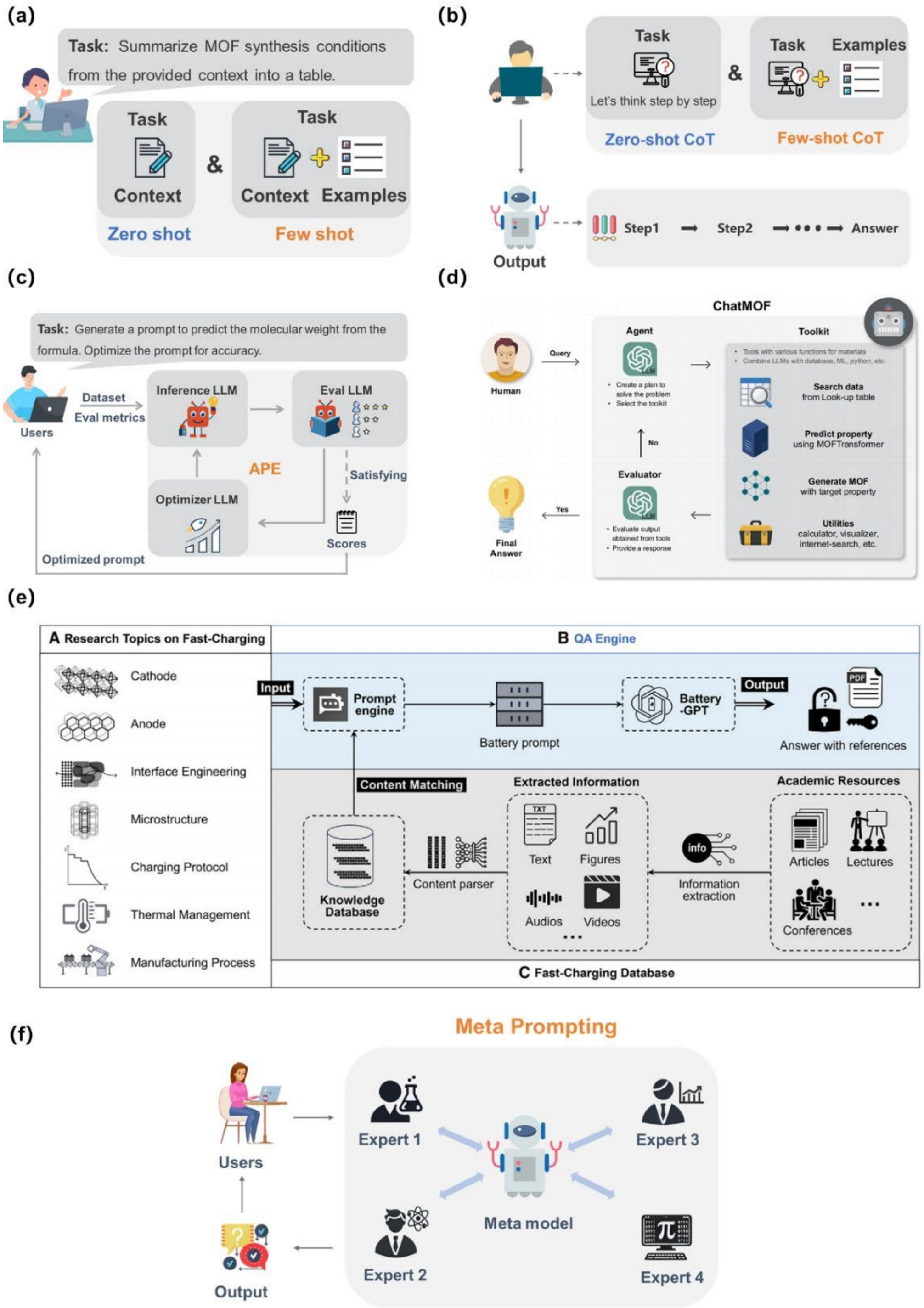

5. Regarding my earlier comment #6, I appreciate that the authors have addressed key limitations, particularly in discussing alternative approaches like directed prediction. If they can include examples to illustrate this, that would be even better, but the current discussion is already a strong improvement. Additionally, it is worth considering how

function-calling or tool using capabilities in LLMs can be leveraged to access external prediction tools (e.g., Python based mathematical calculations, trained ML models, or computational chemistry software). Instead of having LLMs directly generate predictions, they could be prompted to determine appropriate input parameters for external tools, retrieve results, and interpret them. This would be a valuable point to introduce in the discussion of prompt engineering's future applications.

Author Response: We greatly appreciate your thoughtful suggestions on the discussion regarding future research directions regarding prediction tasks. We have further enhanced the manuscript by incorporating examples to illustrate the proposed methods, as well as addressing the potential of leveraging external tools for predictions.

Updates in the Revised Manuscript:

Line 315-317, Page 17, Revised Manuscript: One potential way to mitigate this by designing prompts that convert continuous numerical predictions into discrete interval predictions (e.g., predicting a range of property rather than requesting an exact value).

Line 320-325, Page 17, Revised Manuscript: Furthermore, an alternative approach is integrating external tools like ReAct. Instead of having LLMs generate numerical predictions directly, the models can be prompted to select appropriate external tools to get the results and interpret them. For instance, the LLM could determine the molecular properties for a compound by calling a computational chemistry software to estimate its physical/chemical properties, offering more precise and reliable predictions.

Overall, I see that the manuscript has improved significantly, and I am sure addressing above additional minor points would further enhance its clarity and usefulness to the intended chemistry audience.

Author Response: We are grateful for the reviewer's constructive comments and suggestions.

Reviewer 2

Comments:

The authors have addressed all my comments adequately.

Author Response: We thank the reviewer for the comments again.
